# Deciphering the Crystallinity‐Dependent Sensitivity of Charge Injection in n‐Channel Organic Transistors: Reliable Characterization and Optimized Performance

**DOI:** 10.1002/advs.77050

**Published:** 2026-08-21

**Authors:** Walid Boukhili, Chan Zhang, Jianeng Ma, Changwoo Yu, Chee Leong Tan, Huabin Sun, Zhihao Yu, Guangan Yang, Xiang Wan, Li Zhu, Yong Xu, Liyang Yu, Ruipeng Li, Seung‐Hoon Lee, Zhuping Fei, Dongyoon Khim

**Affiliations:** ^1^ College of Integrated Circuit Science and Engineering Nanjing University of Posts and Telecommunications Nanjing People's Republic of China; ^2^ Institute of Molecular Plus Department of Chemistry Tianjin Key Laboratory of Molecular Optoelectronic Science Tianjin University Tianjin People's Republic of China; ^3^ Division of Advanced Materials and Engineering Kongju National University Chungcheongnam‐do Republic of Korea; ^4^ Guangdong Greater Bay Area Institute of Integrated Circuit and System Guangzhou People's Republic of China; ^5^ Research Institute of Frontier Science Southwest Jiaotong University Chengdu Chengdu People's Republic of China; ^6^ National Synchrotron Light Source (NSLS II) Brookhaven National Laboratory Upton New York USA

**Keywords:** contact resistance, crystallinity of conjugated polymers, environmental and operational stability, low‐temperature measurements, N‐type organic transistors

## Abstract

The performance and reliability of n‐type organic field‐effect transistors (OFETs) are strongly influenced by the interplay between semiconductor crystallinity and the metal/semiconductor interface. Here, we systematically investigate a series of diketopyrrolopyrrole (DPP)‐based n‐type polymers with identical backbones but varying side‐chain architectures, resulting in varied long‐range order. By comparing devices fabricated with conventional Au and low‐work‐function Al/Ti electrodes, we elucidate the synergistic effects of intrinsic polymer crystallinity and interfacial energetics on charge injection, transport, and operational stability. Temperature‐dependent electrical measurements and trap analyses demonstrate that contact resistance, rather than intrinsic bulk transport, dominates the observed mobility differences between polymers of moderate and high crystallinity. Optimized Al/Ti contacts mitigate these interfacial limitations, enabling a transition from contact‐limited to channel‐limited transport and significantly improving environmental and operational stability. These findings establish a quantitative framework linking crystalline order, injection efficiency, and device reliability. This framework provides critical design guidelines for the evaluation and optimization of high‐performance n‐type organic semiconductors.

## Graphical abstract

Precise characterization of n‐type organic semiconductors necessitates decoupling interfacial constraints from intrinsic charge transport. This work reveals that disordered polymers are particularly vulnerable to contact‐limited bottlenecks compared to their crystalline counterparts. By employing Al/Ti electrodes to optimize injection, this research establishes a vital framework for the accurate evaluation and rational design of high‐performance, air‐stable organic electronics.

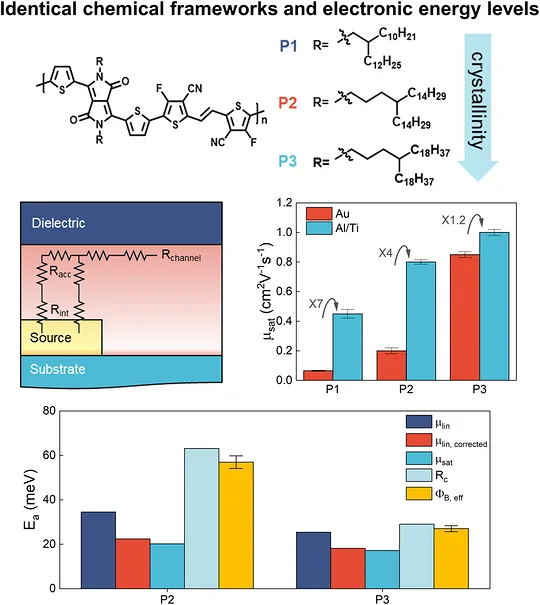

## 1 Introduction

Organic field‐effect transistors (OFETs) have long been recognized not only as building blocks for flexible, low‐cost electronics, but also as essential analytical tools for elucidating fundamental charge transport physics in organic semiconductors [1, 2, 3, 4, 5]. Driven by the rapid development of novel organic semiconductors and deeper insights into device physics, OFET performance has improved remarkably, with recent studies reporting charge carrier mobilities exceeding 10 cm^2^ V^−^^1^s^−^^1^ [6, 7, 8]. Despite decades of progress, the field still struggles with a lack of deterministic guidelines for optimizing device performance, particularly in n‐type systems [9, 10, 11]. Critical limitations in OFET performance often stem from the contact interface [12, 13, 14], where the interplay of energetics and the semiconductor microstructure at the junction modulates charge injection and transport [11, 15, 16]. While the impact of this metal‐semiconductor interface is widely recognized, the sensitivity of charge injection to contact resistance is intricately governed by complex morphological features. This microstructure encompasses not only long‐range macroscopic crystallinity and molecular orientation (e.g., edge‐on vs. face‐on stacking) but also local, short‐range order and aggregate networks [17, 18, 19, 20, 21]. Depending on the specific polymer system, low contact resistance can be achieved either through high crystallinity or via dense, localized short‐range aggregates that mitigate energetic disorder and provide efficient percolation pathways for injected carriers [17, 18, 19, 20, 21]. A prime example is the well‐studied n‐type polymer P(NDI2OD‐T2) (commercially known as N2200); despite adopting a predominantly ‘face‐on’ orientation with out‐of‐plane π‐π stacking, it exhibits remarkable tolerance to high injection barriers owing to its strong solution‐state pre‐aggregation tendency and the resulting dense, short‐range localized aggregate networks [17, 18, 20]. However, despite its critical importance, the precise nature of these interfacial microstructural interactions remains elusive, particularly for electron‐transporting systems. Consequently, this study aims to tackle three critical unresolved issues regarding charge injection that hinder the accurate evaluation and practical application of n‐type organic semiconductors.

First, the efficacy of charge injection engineering remains shrouded in empirical uncertainty. It is well documented that strategies such as inserting dipole interlayers or modifying electrode work functions can drastically improve performance for some materials [22, 23, 24, 25] but yield negligible results for others, even when the semiconductors exhibit nearly identical frontier orbital energy levels, suggesting that additional interfacial or microstructural factors govern charge injection. Currently, interface development relies heavily on empirical optimization [26, 27], as the literature reports performance enhancements without providing a clear physical rationale for the material‐dependent response to injection engineering. There is an urgent need to understand why the impact of charge injection varies so profoundly across different semiconducting platforms.

Second, a significant discrepancy often exists between molecular design and the realized performance of n‐channel polymers [15, 28, 29, 30, 31, 32, 33, 34, 35]. Researchers frequently observe that minor structural modifications, such as side‐chain engineering on an otherwise identical polymer backbone, lead to substantial differences in measured electron mobility [30, 31, 32, 33, 34, 35]. These disparities are commonly attributed to subtle variations in crystalline order; however, this explanation alone is insufficient. In bottom‐contact OFET geometries, variations in contact resistance can artificially amplify apparent mobility differences [12, 13, 36, 37], making it unclear whether performance gaps originate from intrinsic differences in crystalline registry and bulk transport or from extrinsic limitations imposed by the electrode–semiconductor interface. Without rigorously decoupling contact resistance from channel transport, the intrinsic structure–property relationship remains obscured.

Third, the environmental and operational stability of n‐type devices is often fundamentally underrated [38, 39, 40, 41, 42, 43, 44]. While p‐type semiconductors have reached levels of stability sufficient for commercialization [43, 44, 45, 46], n‐type materials remain highly susceptible to electron‐trapping species such as moisture and oxygen [45, 46, 47]. When stability is evaluated in a non‐optimized OFET configuration, the results are frequently compromised by inefficient charge injection, which accelerates degradation [48, 49]. Furthermore, conventional gold (Au, Φ ≈ 5.1 eV) electrodes, standard for p‐type materials, create a large injection barrier to the lowest unoccupied molecular orbital (LUMO) of most n‐type polymers. This fundamental mismatch leads to a severe underestimation of their true reliability [50, 51]. To establish a fair performance hierarchy, an optimized and standardized platform is essential.

In this study, we address these lingering questions by investigating the synergistic relationship between the semiconductor's microstructure and electrode energetics. To achieve this, we selected a series of three diketopyrrolopyrrole (DPP)‐based n‐channel polymers (P1‐P3) that feature identical chemical backbones and nearly identical LUMO levels but exhibit distinct degrees of crystalline order. By systematically comparing their behavior using conventional Au and optimized Al/Ti electrodes, we elucidate how the semiconductor's morphology dictates its susceptibility to contact resistance and environmental stress. While electrode work‐function alignment is a well‐established factor governing charge injection, this study reveals that the sensitivity to contact resistance is disproportionately amplified in disordered polymers. By decoupling these contact effects, we demonstrate that what was previously interpreted as poor intrinsic transport in low‐crystallinity n‐type materials is often an artifact of improper contact engineering, providing a critical guideline for accurate material evaluation.

## 2 Results and Discussion

**FIGURE 1:**
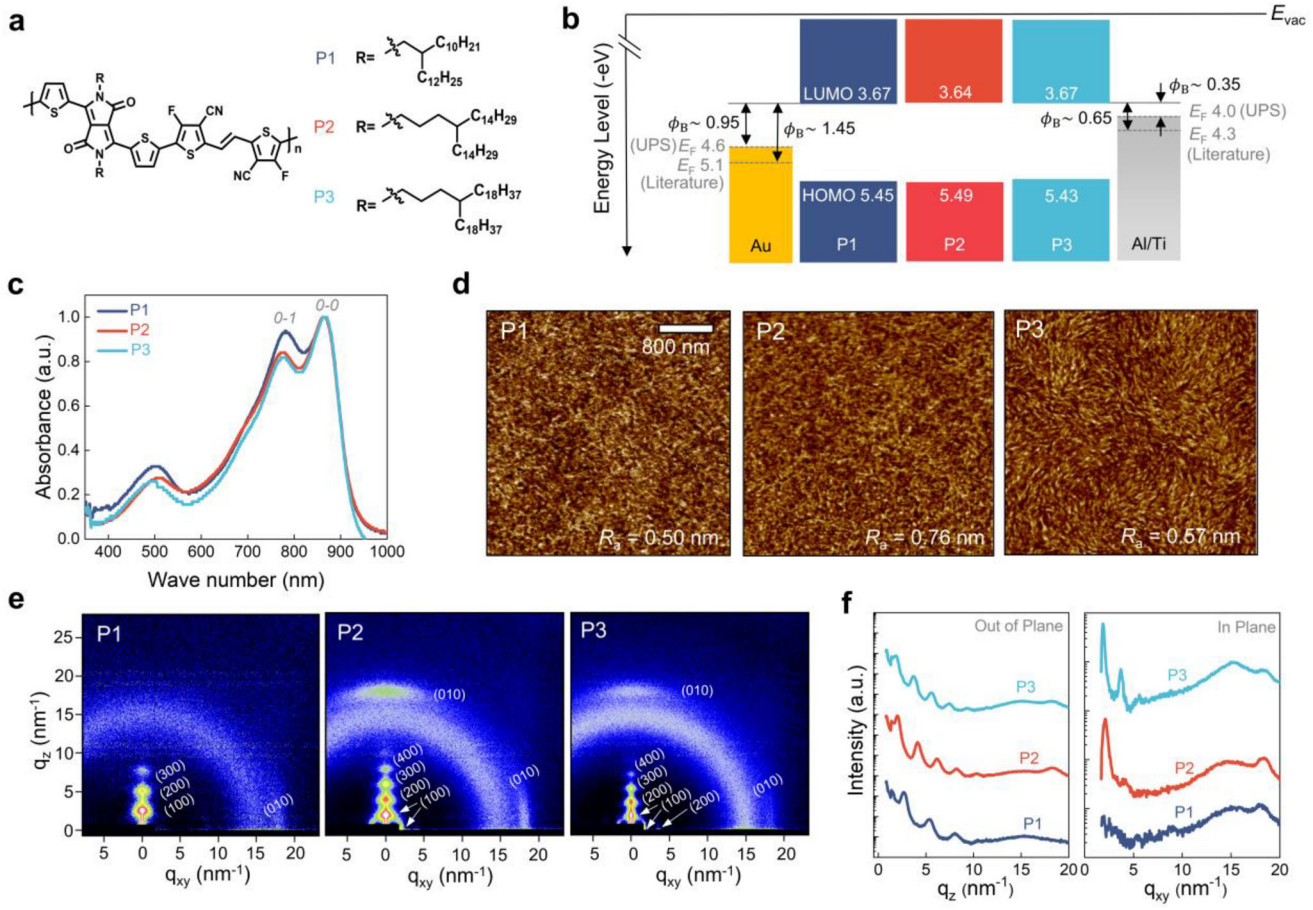
Molecular design and physicochemical characterization of P2F2CNTVT‐DPP derivatives. (a) Chemical structures of the synthesized polymers P1, P2, and P3 with varying side‐chain architectures. (b) Energy level diagram illustrating the HOMO/LUMO levels of the polymers relative to the work functions of Au and Al/Ti electrodes. (c) UV–vis–NIR absorption spectra of the polymer thin films showing vibronic transition features. (d) AFM height images displaying surface morphology and root‐mean‐square (RMS) roughness (R_a_). (e) Two‐dimensional GIWAXS patterns and (f) corresponding one‐dimensional line‐cut profiles in the out‐of‐plane (left) and in‐plane (right) directions, revealing crystalline packing and backbone orientation.

The three diketopyrrolopyrrole (DPP)‐based polymers, P2F2CNTVT‐DPP1 (P1), P2F2CNTVT‐DPP2 (P2), and P2F2CNTVT‐DPP3 (P3), which systematically differ in their branched alkyl substituents (R): P1 (R = 2‐decyltetradecyl), P2 (R = 2‐hexyltetradecyl), and P3 (R = 2‐octyldodecyl) were synthesized and purified following the previously reported procedures to ensure structural consistency (Figure 1a) [30]. P1, P2, and P3 exhibited number‐average molecular weights (M_n_) of 31.2, 37.6, and 36.0 kDa, respectively, with polydispersity indices (Đ) ranging from 2.2 to 3.6. The thermal properties of P1‐P3 were confirmed by thermal gravimetric analysis (TGA) and differential scanning calorimetry (DSC), showing excellent thermal stability up to 400°C and side‐chain melting transitions consistent with prior reports on DPP‐based copolymers [30]. Based on the optimized conditions established in the literature and our own performance validation at various temperatures, 200°C was employed as the optimal annealing temperature to facilitate kinetic reorganization into a highly crystalline state without chemical degradation. Despite these side‐chain modifications, all three polymers exhibited nearly identical electronic structures. As shown in the energy level diagram (Figure 1b), the LUMO levels are closely aligned within a narrow range of −3.64 to −3.67 eV. Given their n‐type character, the estimated electron injection barrier (ϕ_B_) is approximately 0.95 eV for Au and 0.35 eV for Al/Ti electrodes, remaining effectively constant across the three polymers. The HOMO levels also show only marginal variations, with P3 possessing the deepest value of −5.43 eV. Figure 1c presents the normalized UV–vis absorption spectra for the three conjugated polymer thin films. The spectra highlight two main transitions: the 0−0 peak, corresponding to a purely electronic transition from the vibrational ground state to the vibrational ground state of the excited state; and the 0−1 peak, representing a transition from the vibrational ground state to the first vibrationally excited state [52]. The relative intensity ratio of the 0–0 to 0–1 vibronic peaks (A_0_–_0_/A_0_–_1_) serves as a sensitive indicator of intermolecular coupling and aggregate character in the solid state. A higher ratio, as observed for P2 and P3 compared to P1, indicates stronger J‐aggregate character and enhanced molecular ordering [53], which promotes electronic delocalization and more efficient charge transport. This increased ordering typically correlates with improved electronic delocalization along the polymer backbone and leads to more efficient charge transport in devices. Such improved molecular ordering is a prerequisite for high‐performance charge transport [31, 32, 33], suggesting that P2 and P3 possess a more favorable framework for carrier hopping than P1. Crucially, while the A_0_–_0_/A_0_–_1_ ratio varies, the nearly identical absorption onsets across the series confirm that the intrinsic electronic structure and backbone planarity are not significantly compromised by the side‐chain variations.

The surface morphologies and fibrillar networks of the polymer films annealed at 200°C were investigated by tapping‐mode atomic force microscopy (AFM), as shown in Figure 1d. All polymer films exhibited distinct semicrystalline topographies characterized by interconnected, fiber‐like structures. The corresponding root‐mean‐square (RMS) roughness values were measured to be 0.50, 0.76, and 0.57 nm for P1, P2, and P3, respectively, indicating the formation of smooth and morphologically uniform thin films. P1 forms a relatively disordered topography with small, isolated fibrils, which may lead to numerous grain boundaries that impede charge flow. In contrast, P2 and P3 develop highly continuous and interconnected fibrillar networks, with P3 exhibiting the most extensive domain continuity. This morphological refinement is quantitatively supported by grazing‐incidence wide‐angle X‐ray scattering (GIWAXS) analysis.

As shown in the two‐dimensional (2D) scattering patterns and the corresponding one‐dimensional (1D) line‐cut profiles (Figure 1e,f), all three polymers exhibit a predominant edge‐on orientation, as indicated by the strong (h00) lamellar diffraction peaks along the out‐of‐plane (q_z_) direction and the distinct (010) π–π stacking peak along the in‐plane (q_xy_) direction. The degree of long‐range structural order displays a clear dependency on the side‐chain length [46]; extending the alkyl chain leads to an increase in the out‐of‐plane lamellar d‐spacing from 24.1 to 34.1 Å, while simultaneously inducing a contraction in the in‐plane π‐π stacking distance from 3.48 to 3.41 Å for the dominant edge‐on crystallites. Interestingly, the increased side‐chain length also facilitates the formation of a minority face‐on population, as indicated by the emergence of in‐plane (h00) and out‐of‐plane (010) diffraction signals [54]. The enhanced crystallinity in the P1‐P3 series stems from the strategic optimization of side‐chain length and branching architecture. Increasing the alkyl chain length to C18 in P3 intensifies van der Waals interactions and hydrophobic driving forces, facilitating robust molecular self‐assembly during film formation. This is evidenced by high‐order (h00) diffraction peaks and a pronounced (010) π–π stacking intensity in GIWAXS, indicating superior long‐range lamellar ordering. Furthermore, optimizing the branching position minimizes steric hindrance near the polymer backbone. This structural refinement promotes a highly ordered ‘edge‐on’ orientation and increases the crystal coherence length (L_c_), thereby enhancing the structural quality of the semiconducting layer [24]. Consequently, the C_18_ side chains in P3 serve as an internal plasticizer that enables kinetic reorganization into a highly crystalline state. Collectively, the structural characterization, centered on the GIWAXS results corroborated by the well‐defined continuous fibrillar networks observed in AFM, reveals a compelling dichotomy: while P1, P2, and P3 share nearly identical chemical frameworks and electronic energy levels, they exhibit a clear hierarchy in their long‐range ordering and crystalline quality in the order of P3> P2> P1. This unique combination of properties establishes this polymer series as a robust platform for investigating the complex interplay between intrinsic crystallinity and interfacial contact effects.

**FIGURE 2:**
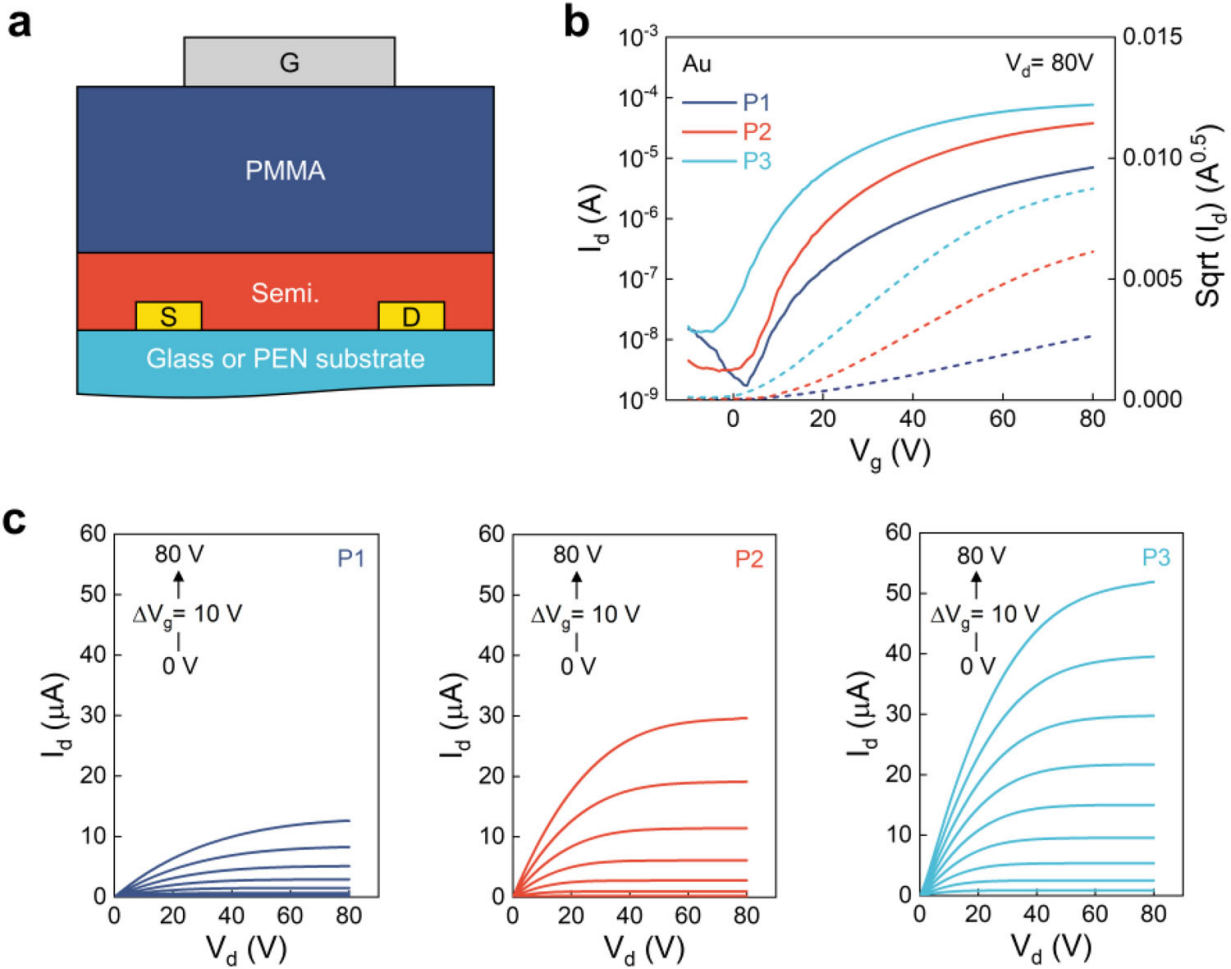
Device structure and electrical performance of OFETs with Au electrodes. (a) Schematic of the top‐gate/bottom‐contact (TG/BC) transistor architecture. (b) Representative transfer characteristics of P1, P2, and P3 measured in the saturation regime at V_d_ = 80 V. Solid and dashed lines correspond to I_d_ (semi‐log scale) and square root of I_d_, respectively. (c) Representative output characteristics of P1, P2, and P3 devices, with Vg increasing from 0 to 80 V.

To evaluate the charge transport capabilities, top‐gate/bottom‐contact (TG/BC) organic field‐effect transistors (OFETs) were fabricated using Au source/drain electrodes and a PMMA dielectric (Figure 2a). The representative transfer and output characteristics (Figure 2b,c) demonstrate robust n‐type operation for all three polymers. The key electrical parameters of the Au‐contact OTFTs based on P1–P3 are summarized in Table 1. The device performance exhibits a clear dependency on the side‐chain‐induced crystallinity, following the trend of P1< P2< P3. P1 delivers the weakest performance, with a maximum on‐current (I_on, max_) of 7 × 10^−6^ A and a saturation electron mobility (µ_sat_) of 0.065 (±0.0035) cm^2^V^−^^1^s^−^^1^. P2 shows a moderate improvement, achieving I_on, max_ of 3.5 × 10^−5^ A and µ_sat_ of 0.20 (±0.02) cm^2^V^−^^1^s^−^^1^, which represents an approximately threefold increase in µ_sat_ compared to P1. The highest performance is observed in P3, which reaches an I_on, max_ of 7.6 × 10^−5^ A and µ_sat_ of 0.85 (±0.02) cm^2^V^−^^1^s^−^^1^, nearly 13 times higher than P1. While the overall performance trend correlates well with the crystallinity observed in AFM and GIWAXS analysis, the significant fourfold disparity between P2 and P3 (0.20 vs. 0.85 cm^2^V^−^^1^s^−^^1^) requires further investigation to understand its origin. According to the GIWAXS and AFM data, P2 already possesses a high degree of structural order and a well‐developed fibrillar network, which would typically be sufficient for efficient charge transport [55]. This suggests that the substantial mobility gap between P2 and P3 does not arise solely from intrinsic bulk transport differences associated with crystallinity but is likely amplified by extrinsic interfacial injection limitations.

**TABLE 1:** Summary of key device parameters of OTFTs based on P1–P3 polymers with Au contacts. Values represent the average of 10 devices per polymer/Au‐contact configuration.

Polymer	Mobility (cm^2^V^−^^1^s^−^^1^)	V_on_ (V)	V_th_ (V)	I_on, max_ (A)	SS (mV dec^−^^1^)	D_it_ (cm^−^^2^ eV^−^^1^)
P1	0.065 ± 0.0035	3.06	16 ± 0.5	7.0 × 10^−^^6^	255.63	7.20 × 10^10^
P2	0.20 ± 0.02	1.0	11 ± 0.3	3.5 × 10^−^^5^	115	2.03 × 10^10^
P3	0.85 ± 0.02	−4.5	2.5 ± 0.5	7.6 × 10^−^^5^	100.78	1.74 × 10^10^

**FIGURE 3:**
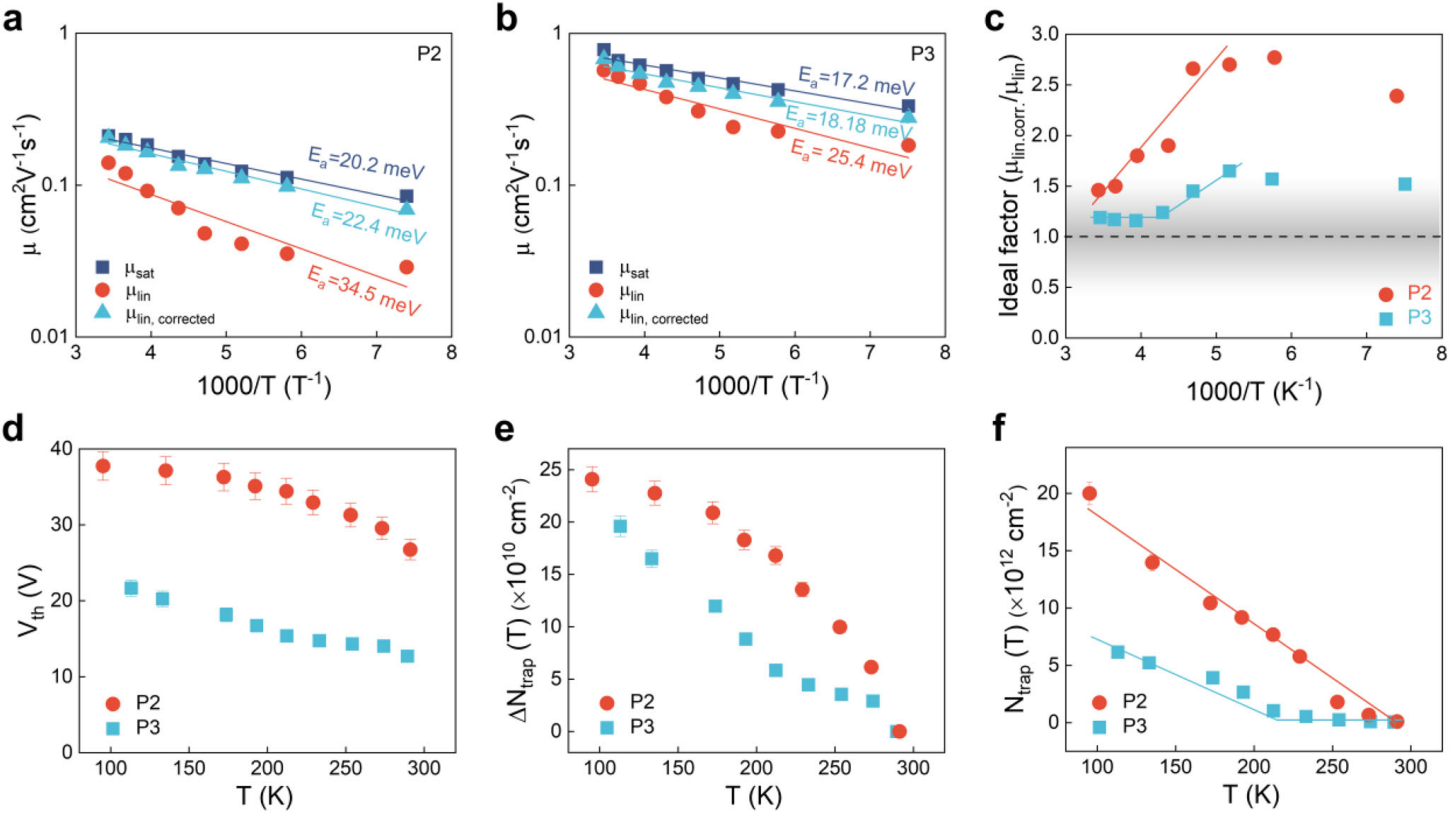
Low temperature‐dependent charge transport and trap analysis. Arrhenius plots of field‐effect mobilities (µ_sat_, µ_lin_, and µ_lin, corrected_) for (a) P2 and (b) P3, with activation energies (E_a_) are extracted from the linear fits. (c) mobility ideal factor, defined as µ_lin, corrected_/µ_lin_, as a function of 1000/T. Temperature‐dependent evolution of (d) threshold voltage (V_th_), (e) change in trap density (ΔN_trap_ (T)), and (f) total trapped charge density (N_trap_ (T)) for P2 and P3 devices.

To further elucidate the underlying charge transport mechanisms and the origin of the performance discrepancy between P2 and P3, we conducted temperature‐dependent measurements. P1 was excluded from this analysis due to its significantly lower crystallinity. Representative linear and saturation transfer characteristics measured across a temperature range of 95 to 289 K are provided in Figure S2. The Arrhenius plots of charge mobility for P2 and P3 (Figure 3a,b) reveal that both polymers exhibit typical thermally activated transport behavior. Interestingly, a significant discrepancy in activation energy (E_a_) was observed between the two polymers in the linear regime (µ_lin_), whereas this gap narrowed considerably in the saturation regime (µ_sat_), highlighting the dominance of contact effects in µ_lin_. Specifically, the E_a_ for µ_lin_ was measured at 34.5 meV for P2 and 25.4 meV for P3. In contrast, the values for µ_sat_ were much more comparable, at 20.2 meV for P2 and 17.2 meV for P3. This regime‐dependent disparity in E_a_ provides critical insights into the interplay between interfacial and bulk transport properties. In the saturation regime, the high drain voltage (V_d_) effectively mitigates the relative impact of contact resistance (R_c_), allowing the extracted mobility to more closely reflect the intrinsic hopping transport within the semiconductor bulk. Consequently, the comparable saturation E_a_ values suggest that the intrinsic energetic landscapes governing bulk hopping transport in P2 and P3 are similar. Conversely, the linear regime is characterized by low V_d_, where the voltage drop across the electrode‐semiconductor interface dominates [56, 57]. The markedly higher E_a_ (34.5 meV) observed for P2 in the linear regime indicates a more pronounced charge injection barrier at the contacts. This implies that the elevated activation energy in P2 is not an inherent property of the polymer matrix itself, but rather a result of a less optimized interfacial contact compared to P3. This interpretation is further validated by the corrected linear mobility (µ_lin, corrected_). A detailed description of the extraction and calculation procedures for the µ_lin, corrected_ is provided in Section S2. After de‐embedding the effects of R_c_, the E_a_ values for P2 and P3 converge toward 22.4 and 18.18 meV, respectively, aligning closely with their saturation counterparts. To quantitatively evaluate the impact of R_c_ on device operation, we analyzed the ‘mobility ideal factor’, defined as the ratio of corrected to uncorrected linear mobility (µ_lin, corrected_/µ_lin_), as a function of temperature (Figure 3c and Section S2). For both polymers, the mobility ideal factor exceeds unity and increases as the temperature decreases, indicating that R_c_ significantly limits the apparent charge transport at lower temperatures. Notably, P2 exhibits a substantially higher mobility ideal factor compared to P3 across the entire temperature range. For instance, at lower temperatures, the mobility ideal factor for P2 rises sharply toward ∼2.7, whereas P3 maintains a relatively lower value of approximately 1.5. This pronounced difference in the mobility ideal factor validates our hypothesis that the discrepancy in E_a_ originates primarily from interfacial effects rather than inferior bulk transport capabilities. While the saturation E_a_ values confirm that the intrinsic hopping barriers are comparable, the higher mobility ideal factor in P2 confirms that its linear regime performance is more severely bottlenecked by a non‐ideal electrode‐semiconductor interface. These findings highlight that P3 not only possesses superior intrinsic transport due to its optimized morphology but also benefits from a much more efficient charge injection environment, which collectively leads to its significantly higher device performance compared to P2.

Temperature‐dependent threshold voltage (V_th_) and the corresponding trap densities were calculated to systematically probe the charge transport physics of P2 and P3. As the temperature decreased from 300 to 95 K, both conjugated polymers exhibited a positive V_th_ shift, consistent with trap‐limited transport models, such as the Multiple Trap and Release (MTR) model [58, 59, 60, 61]. However, a striking divergence in the V_th_ evolution was observed; while P2 showed a continuous and steep increase in V_th_, P3 displayed two distinct regimes. Specifically, between 300 and 200 K, the V_th_ shift for P3 was negligible (from 12.7 to 15.3 V, ΔV_th_ = 2.6 V), whereas P2 exhibited a much more pronounced shift (from 26.7 to 34.4 V, ΔV_th_ = 7.7 V). To quantitatively analyze this behavior, the total trapped charge density (N_trap_ (T)) was determined by integrating the room‐temperature trap density extracted from the subthreshold swing (SS) with the temperature‐dependent variation (ΔN_trap_ (T)) derived from ΔV_th_ [60, 62, 63]:

(1) $$N_{SS}^{\max}=\frac{C_{i}}{q}\;\left[\frac{SS\;}{ln\left(10\right)k_{B}T/q}-1\right]$$

The temperature‐dependent trapped charge variation, ΔN_trap_, can be obtained from ΔV_th_ as [60, 62, 63]:

(2) $$\Delta N_{trap}\;\left(T\right)=\frac{C_{i}\Delta V_{th}\left(T\right)\;}{q}$$

allowing the total trapped charge density, N_trap_ (T), to be determined at each temperature. As illustrated in Figure 3e, the ΔN_trap_ (T) for P2 increased monotonically to 16.8 × 10^10^ cm^−2^ at 200 K, while P3 showed a remarkably suppressed increase of only 5.8 × 10^10^ cm^−2^. This trend is even more evident in the total N_trap_ (T) profiles in Figure 3f; for P2, N_trap_ (T) rose continuously to 20 × 10^12^ cm^−2^ at 113 K. In contrast, P3 maintained a significantly lower trap density of approximately 1 × 10^12^ cm^−2^ down to 200 K, before linearly increasing to 7.6 × 10^12^ cm^−2^ at 113 K. These quantitative findings reveal that highly crystalline P3 exhibits significantly reduced susceptibility to deep trap formation that would otherwise bottleneck charge transport at ambient temperature [64]. Conversely, for the lower‐crystalline P2, the immediate and sustained escalation in trap density upon cooling signifies that a deep‐trapping regime, stemming from reduced molecular ordering, remains dominant even at 300 K. This interpretation is further corroborated by the ideal factor trends in Figure 3c, where P3 maintains a constant value down to approximately 200 K, directly mirroring its stable V_th_ characteristics and superior crystalline ordering. A more detailed analysis of low‐temperature electrical behavior and trap states in top‐gate/bottom‐contact OFETs (P2 and P3) is provided in Section S4, focusing on V_th_, N_trap_, drain current (I_d_), and its temperature dependence (activation energy, E_a_), as well as the density of states (DOS) of shallow and deep traps.

While deep traps in OFETs generally arise from structural disorder at grain boundaries, chemical impurities (e.g., moisture and oxygen), and interfacial states at the dielectric boundary, the role of the semiconductor/metal contact is equally critical [65]. Ultimately, as evidenced by the mobility‐dependent activation trends in Figure 3a–c, the disparity in R_c_ between P2 and P3, which is associated with differences in their microstructural organization and degree of crystalline order, rather than purely intrinsic bulk traps, is considered the primary driver of the observed performance variations.

**FIGURE 4:**
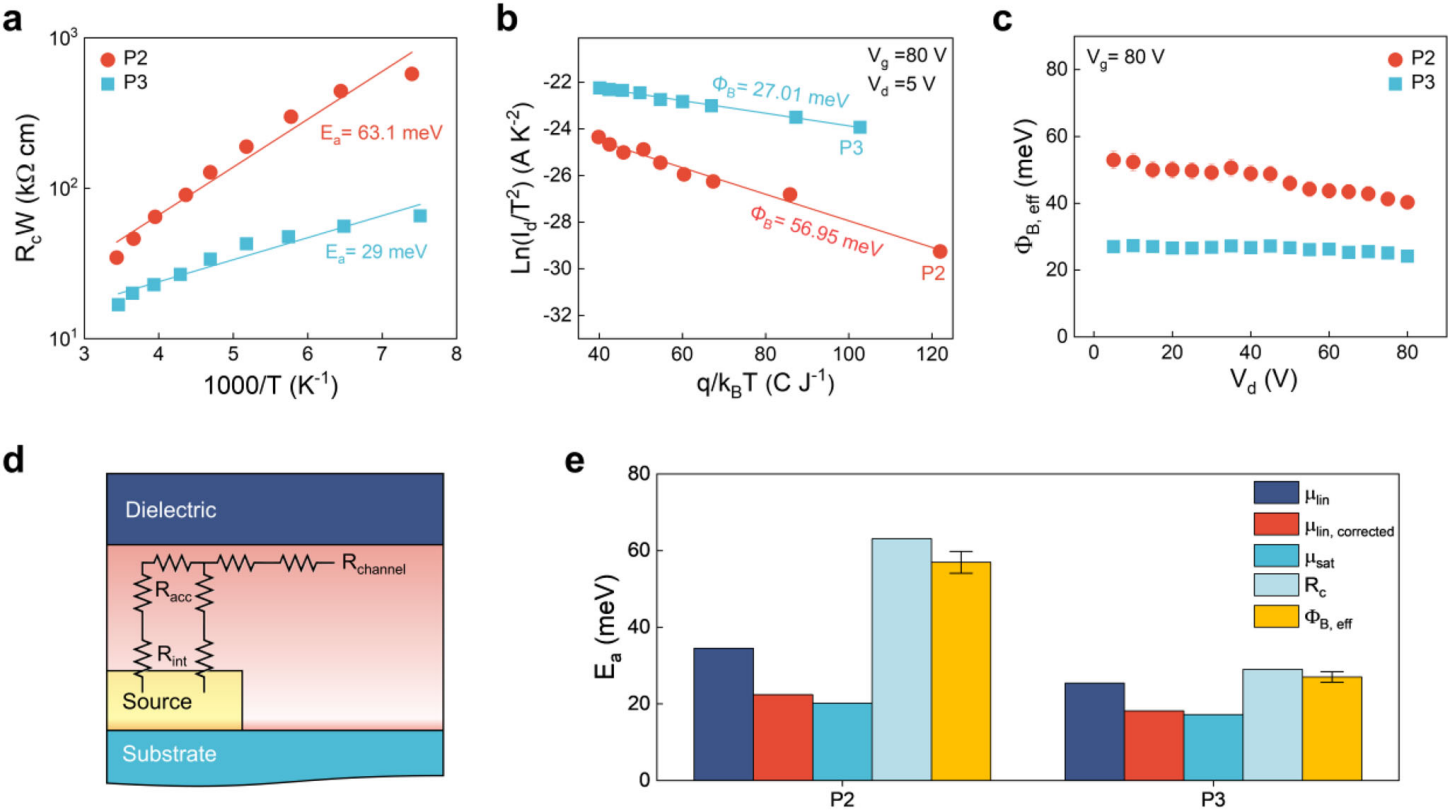
Contact resistance and charge injection analysis. (a) Temperature dependence of width‐normalized contact resistance (R_c_W) for P2 and P3, with extracted activation energies (E_a_). (b) Richardson plots (ln(I_d_/T^2^) vs. q/(k_B_T)) used to determine the injection barrier height (ϕ_B_). (c) Effective Schottky barrier height of P2 and P3 OFETs as a function of V_d_, showing lower and more stable injection barriers for P3. (d) Schematic diagram of the resistance components in TG/BC OFETs at the metal–semiconductor interface, including interface (R_int_), access (R_acc_), and channel (R_channel_) resistances. (e) Comparison of E_a_ for various parameters (µ_lin_, µ_lin, corrected,_ µ_sat_, R_c_, and ϕ_B, eff_) between P2 and P3.

To further investigate the quantitative influence of R_c_ in P2 and P3, we analyzed its temperature dependence, which serves as a direct indicator of the total energetic barrier for charge injection. As illustrated in Figure 4a, the R_c_ for both polymers exhibits a monotonically increasing trend with decreasing temperature. This behavior is fundamentally consistent with the thermally activated hopping transport mechanism characteristic of organic semiconductors, confirming that R_c_ plays a non‐negligible role in governing the overall charge transport [61]. Specifically, the activation energy of E_a_ (R_c_) for P2 was calculated to be 63.1 meV, whereas P3 exhibited a significantly lower value of 29 meV. This pronounced disparity suggests that charge carriers in P2 encounter a much more formidable energetic barrier during their transition from the electrode into the conducting channel, thereby inducing a severe contact‐limited transport regime. The effective Schottky barrier height (Φ_B_, _eff_) was extracted from the Arrhenius‐type plots of ln (I_d_/T^2^) vs. q/(k_B_T) (Figure 4b), utilizing data obtained from the output characteristics (see Figure S5 and Section S4). The estimated Φ_B_, _eff_ as a function of drain voltage (V_d_) for both samples is presented in Figure 4c. Remarkably, P2 exhibited a Φ_B_, _eff_ of 57 meV, which is substantially higher than the 27 meV observed for P3. In classical theory, Φ_B_, _eff_ is primarily determined by the energetic mismatch between the semiconductor's LUMO level and the electrode's work function. However, despite P2 and P3 possessing nearly identical LUMO levels, a significant discrepancy in their effective injection barriers arises, leading to vastly different R_c_. In the framework of OFET physics, R_c_ is modeled as the summation of the intrinsic metal/semiconductor interfacial resistance (R_int_) and the access resistance (R_acc_) of the semiconductor bulk adjacent to the contacts, as shown in Figure 4d [66]. While R_int_ is governed by Φ_B_, _eff_, R_acc_ is dictated by local crystallinity, film thickness, and energetic disorder. Consequently, Φ_B_, _eff_ represents the barrier at the primary interface R_int_ while implicitly accounting for the bulk transport contribution (R_acc_), such that Φ_B, eff_ ≤ E_a_ (R_c_) since R_c_ includes both interfacial barrier and thermally activated bulk access resistance [66]. For P3, the proximity of E_a_ (R_c_) ∼ 29 meV and Φ_B, eff_ ∼ 27 meV indicates a minimal R_acc_ due to its high crystallinity. In contrast, the larger disparity in P2, where E_a_(R_c_) ≈ 63 meV and Φ_B, eff_ ≈ 57 meV, confirms that access resistance significantly bottlenecks charge injection in the lower‐crystallinity polymer. The larger R_acc_ observed for P2 is attributed to its less ordered microstructure in the semiconductor region adjacent to the contacts. Reduced crystallinity and increased structural disorder can disrupt intermolecular π–π stacking and hinder the formation of efficient charge‐percolation pathways for injected carriers, leading to additional transport resistance before carriers reach the accumulated channel. By contrast, the more ordered microstructure of P3, characterized by a higher degree of crystalline order, is expected to facilitate more continuous transport pathways near the contact interface, thereby suppressing R_acc_. Furthermore, as illustrated in Figure 4c, the Φ_B, eff_ of P2 exhibits a significant downward trend with increasing V_d_, falling from approximately 55 meV (V_d_ = 5 V) to 40 meV (V_d_ = 80 V). This behavior is primarily attributed to the field‐induced barrier‐lowering effect [60], where the intensified lateral electric field assists charge injection across the high interfacial barrier of the lower‐crystallinity P2 polymer. On the contrary, P3 maintains a remarkably constant Φ_B, eff_ of ∼27 meV, demonstrating exceptional stability regardless of the V_d_ magnitude. This voltage‐independent characteristic suggests that P3 has already achieved a near‐ideal, transparent injection interface, facilitated by its superior crystalline registry and significantly minimized trap density. Consequently, the charge transport in P3 remains largely insensitive to field‐assisted injection, whereas the performance of P2 continues to be heavily governed by its inherent contact‐limited nature.

Figure 4e summarizes the E_a_ for the various parameters discussed, including µ_lin,_ µ_lin, corrected_, µ_sat,_ R_c_, and Φ_B, eff_. This comprehensive evaluation demonstrates that the performance disparity between P2 and P3 stems primarily from interfacial and access‐region energetics rather than intrinsic bulk transport properties. Specifically, both polymers share similar intrinsic transport barriers (∼20 meV of µ_lin, corrected_ and µ_sat_); however, P2 exhibits significantly higher E_a_ values for µ_lin_, R_c_, and Φ_B, eff_, indicating a much more resistive injection environment. These findings demonstrate that despite their nearly identical energy levels, the distinct crystalline registry of these semiconductors leads to a profound difference in the ‘perceived’ injection barrier within the device, serving as the primary origin for the observed divergence in charge carrier mobility.

**FIGURE 5:**
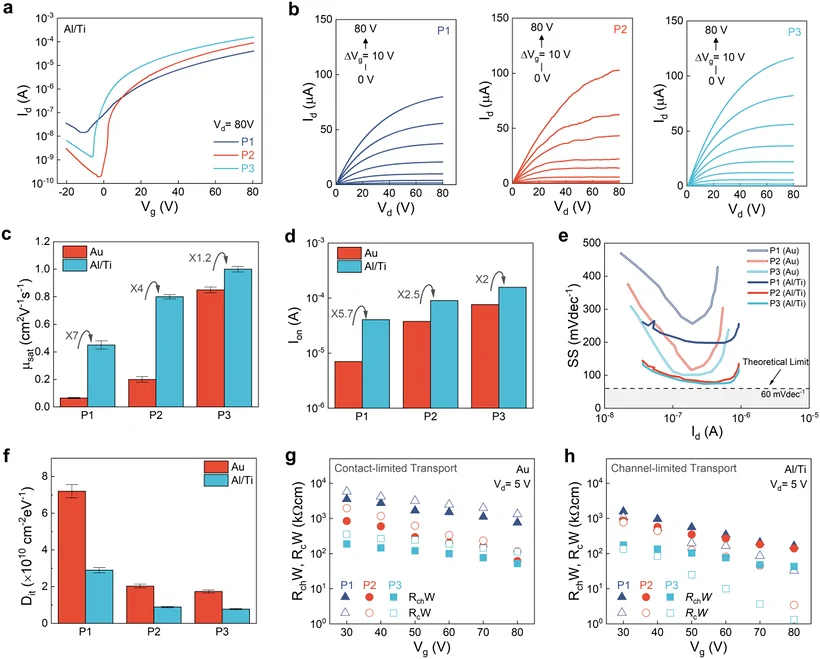
Comparative analysis of electrode effects on OFET performance. (a) Representative transfer and (b) output characteristics of TG/BC OFETs with Al/Ti electrodes. Comparison of (c) µ_sat_ and (d) on‐current (I_on_) for Au (red) and Al/Ti (blue) electrodes across P1, P2, and P3 OFETs. (e) Subthreshold swing (SS) vs. I_d_, highlighting the proximity of Al/Ti‐based P2 and P3 OFETs to the theoretical limit (60 mV dec^−^^1^). (f) Interface trap density (D_it_) for Au and Al/Ti electrodes across P1, P2, and P3 OFETs. (g,h) Comparison of channel resistance (R_ch_W) and contact resistance (R_c_W) for (g) Au and (h) Al/Ti electrodes. Transition from contact‐limited transport (Au) to channel‐limited transport (Al/Ti) is observed for all polymers.

Next, we investigated how the device performance of P1, P2, and P3 evolves when the theoretical injection barrier is reduced. In n‐type OFETs, minimizing the energetic mismatch between the semiconductor LUMO level and the electrode work function is essential for suppressing the charge injection barrier [12, 13, 67, 68]. In this study, we implemented Al/Ti electrodes, which exhibit a low work function of approximately 4.0 eV as confirmed by UPS, while maintaining superior stability. This electrode modification provides a significantly reduced injection barrier of approximately 0.35 eV for the P1‐P3 series, a substantial improvement over the ∼1.0 eV mismatch characteristic of conventional Au electrodes. The representative transfer and output characteristics of the TG/BC OFETs utilizing Al/Ti contacts are depicted in Figure 5a,b. A comprehensive summary of the key electrical parameters for P1–P3 OFETs employing Al/Ti contacts is provided in Table 2. All devices integrated with Al/Ti electrodes demonstrated markedly enhanced electron drain currents (I_d_) and more idealized transfer characteristics, specifically characterized by suppressed off‐state currents in P2 and P3. The corresponding output curves corroborate these improvements, exhibiting distinct linear behavior at low drain voltages, which is indicative of efficient, Ohmic‐like charge injection, and well‐defined current saturation at higher V_d_, consistent with high‐quality semiconducting transport. A compelling trend emerges when analyzing the degree of performance enhancement across the polymer series, as summarized in Figure 5c,d. Although the theoretical reduction in the Schottky barrier height was uniform for all polymers, the magnitude of the resulting performance gain exhibited a clear inverse correlation with the semiconductor's initial crystallinity. For the least crystalline polymer, P1, the transition from Au to Al/Ti contacts resulted in a dramatic sevenfold increase in mobility (from 0.065 to 0.45 cm^2^V^−^^1^s^−^^1^) and a 5.7‐fold enhancement in on‐current. P2 followed this trend with a fourfold mobility increase (from 0.2 to 0.80 cm^2^V^−^^1^s^−^^1^) and a 2.5‐fold gain in on‐current. In contrast, the highly crystalline P3 showed the most modest sensitivity to contact modification, with mobility increasing only 1.2‐fold (from 0.85 to 1.0 cm^2^V^−^^1^s^−^^1^) and on‐current rising twofold. These results suggest that the disordered P1 polymer was initially heavily bottlenecked by its contact resistance, whereas P3 was already operating near its intrinsic transport limit even with less optimized contacts. Furthermore, the implementation of Al/Ti contacts led to significant improvements in subthreshold swing (SS) and interface trap density (D_it_), as illustrated in Figure 5e,f. The SS values for P1‐P3 decreased markedly to 198, 78.6, and 75.3 mV dec^−^^1^, respectively, with P2 and P3 approaching the theoretical thermal limit of 60 mV dec^−^^1^ for trap‐free interfaces at room temperature. This enhanced gate tunability is directly linked to a systematic suppression of interface trap densities [16, 69]. Specifically, with Al/Ti contacts, D_it_ values were significantly reduced to approximately 2.9 × 10^10^ cm^−2^eV^−1^ for P1, 8.9 × 10^9^ cm^−2^eV^−1^ for P2, and 7.8 × 10^9^ cm^−2^eV^−1^ for P3. These reductions represent improvements by factors of approximately 2.5 ×, 2.3 ×, and 2.2 × for P1, P2, and P3, respectively. Collectively, these findings confirm that while Al/Ti contacts universally enhance gate‐coupling efficiency and suppress trap densities, the most profound impact is observed in polymers with lower crystalline order, where contact‐limited transport is most prevalent.

**TABLE 2:** Summary of key device parameters of OFETs based on P1–P3 polymers with Al/Ti contacts. Each value represents the average of 10 devices per polymer/Al/Ti contact configuration.

Polymer	Mobility (cm^2^V^−^^1^s^−^^1^)	V_on_ (V)	V_th_ (V)	I_on, max_ (A)	SS (mV dec^−^^1^)	D_it_ (cm^−^^2^ eV^−^^1^)
P1	0.45 ± 0.03	−10.5	7 ± 0.5	4.06 × 10^−^^5^	198	2.9 × 10^10^
P2	0.80 ± 0.015	−1.35	5 ± 0.45	9.01 × 10^−^^5^	78.6	8.9 × 10^9^
P3	1.0 ± 0.02	−6.5	0 ± 0.53	1.57 × 10^−^^4^	75.3	7.8 × 10^9^

The comparative analysis of width‐normalized contact resistance (R_c_W) and channel resistance (R_ch_W) for OFETs based on Au and Al/Ti electrodes provides clear evidence of the transport regimes governing these devices, as illustrated in Figure 5g,h, Figures S1 and S3. A highly distinctive behavior is observed in the devices utilizing conventional Au electrodes; specifically, R_c_W remains consistently higher than R_ch_W across the entire measured gate voltage (V_g_) range for P1, P2, and P3. This clear dominance of contact resistance signifies a contact‐limited transport regime, where the extrinsic energetic barrier at the metal‐semiconductor interface—rather than the intrinsic charge‐carrying capability of the polymer—dictates the overall device performance. In stark contrast, the implementation of low‐work‐function Al/Ti electrodes induces a dramatic reduction in R_c_W, effectively shifting the bottleneck of charge transport. In this configuration, R_ch_W becomes the dominant resistive component, facilitating a transition to a channel‐limited transport regime. Under these optimized conditions, the devices more accurately reflect the intrinsic mobility of the n‐type polymers, revealing that when R_c_ is sufficiently minimized, the performance gap between the polymers narrows significantly. Notably, P2 exhibits a mobility and current level remarkably close to those of the highly crystalline P3, suggesting that its potential was previously masked by severe injection inefficiency. These results provide several profound insights into the fundamental evaluation of n‐type organic semiconductors. Conventionally, Au has served as a ubiquitous reference electrode in OFET research; however, while its work function (∼5.1 eV) is well‐aligned with the HOMO levels of most p‐type materials [50, 51], it is fundamentally ill‐suited for n‐type semiconductors with LUMO levels typically ranging between 3.5 and 4.0 eV. Our findings suggest that relying solely on Au contacts can lead to a severe underestimation of an n‐type material's intrinsic performance, particularly for polymers with lower crystallinity. This discrepancy introduces significant challenges in establishing a fair and accurate performance hierarchy among newly synthesized materials. In the case of the P1‐P3 series, their true semiconducting capabilities were only fully revealed through the adoption of Al/Ti electrodes, which represent a move toward “appropriate” contact engineering rather than a mere device modification. Furthermore, this study establishes a critical guideline for the application of interface engineering, such as the insertion of interlayers to modulate charge injection [24, 25, 26, 27]. Despite possessing similar LUMO levels, the sensitivity of these semiconductors to interface optimization appears to be strongly influenced by differences in their microstructural organization, particularly their degree of crystalline order. To experimentally validate this hypothesis, P1–P3 transistors were fabricated by incorporating a well‐documented barium hydroxide (Ba(OH)_2_) as an n‐type interlayer onto Au source/drain electrodes [70, 71]. Detailed information regarding the device architecture, Au/Ba(OH)_2_ interface characterization, and the complete set of transfer characteristics is available in Section S6. Morphological analysis revealed that the Ba(OH)_2_ formed aggregated nanoscale domains on the Au surface, effectively modulating the electrode work function to approximately 4.1 eV (Figure S7c).

Consistent with our hypothesis, insertion of this interlayer led to an enhancement in electrical performance across all OFETs. Intriguingly, the magnitude of this improvement was found to be strongly dependent on the polymer microstructure, with the most pronounced enhancements observed in the less crystalline systems (Figure S7d and Table S6). Specifically, upon the integration of Ba(OH)_2_, the mobility of P1 increased 6.6‐fold to 0.43 cm^2^V^−^^1^s^−^^1^ relative to the bare Au electrode, while P2 and P3 exhibited mobilities of 1.0 cm^2^V^−^^1^s^−^^1^ (a fivefold increase) and 1.1 cm^2^V^−^^1^s^−^^1^ (a 1.3‐fold increase), respectively. The trends observed with Ba(OH)_2_‐modified Au contacts closely mirror those obtained with Al/Ti electrodes, where larger performance gains are found in the less crystalline polymers. These results confirm that when energy level alignment is comparable, charge injection is not primarily energy‐limited but instead governed by microstructure‐dependent contact resistance, as evidenced by the substantially larger performance gains observed in the lower‐crystallinity polymers following contact engineering.

Highly crystalline materials like P3 exhibit a degree of robustness against work function mismatches, making them less sensitive to the specific electrode environment or the addition of interlayers. In contrast, low‐crystallinity systems like P1 are highly susceptible to contact environment fluctuations, where even minor improvements in the injection barrier can lead to orders‐of‐magnitude enhancements in mobility. This crystallinity‐dependent sensitivity suggests that disordered polymers require intensive interface engineering to overcome their inherent contact bottlenecks, whereas ordered systems are more resilient. Ultimately, these findings underscore that the accurate assessment and optimization of n‐type OFETs must account for the intricate interplay between semiconductor microstructure, including crystalline morphology, and the energetic landscape of the contact interface.

**FIGURE 6:**
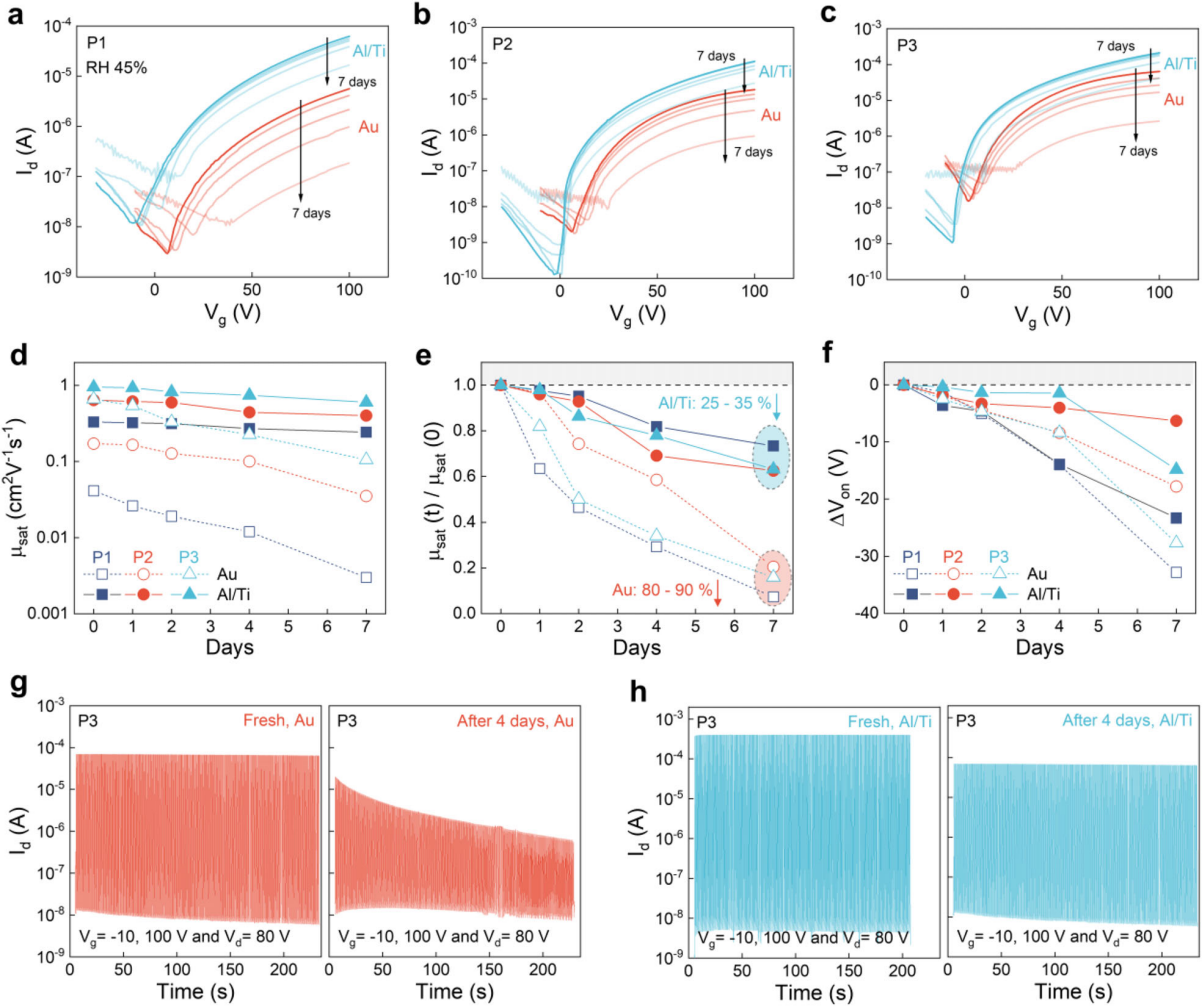
Environmental and operational stability of OFETs. Time‐dependent transfer characteristics of (a) P1, (b) P2, and (c) P3 devices with Au and Al/Ti electrodes, measured over a 7‐day period under ambient conditions at 45% RH. Evolution of (d) saturation mobility (µ_sat_), (e) normalized mobility (µ_sat_(t)/µ_sat_(0)), and (f) turn‐on voltage shift (ΔV_on_) as a function of storage time. Al/Ti‐based OFETs (solid symbols) exhibit significantly higher retention compared to Au‐based OFETs. Operational stability tests for P3‐based OFETs with (g) Au and (h) Al/Ti electrodes. Comparison between fresh and aged (4 days) states under pulsed bias stress (V_g_ = ‐10 to 100 V, V_d_ = 80 V) demonstrates the superior durability of the Al/Ti interface.

Subsequently, we assessed the impact of R_c_ reduction on environmental stability, operational reliability, and overall device behavior. In general, n‐type organic semiconductors are notoriously susceptible to rapid performance degradation upon exposure to ambient air, as moisture and oxygen facilitate the formation of electron traps [45, 46, 47]. Our results demonstrate that the nature of the metal/semiconductor contact strongly influences both the environmental and operational stability of OFETs, likely by suppressing interfacial trap formation and reducing electric‐field‐driven chemical reactions at the contact edge, suggesting that contact engineering is a critical factor in long‐term reliability. To evaluate this, we assessed the stability of P1‐P3 devices over seven days under ambient conditions at an elevated relative humidity (RH) of approximately 45%. The representative transfer characteristics of P1‐P3 OFETs with Au and Al/Ti contacts, measured from day 0 (fresh) to day 7, are presented in Figure 6a–c. All devices utilizing conventional Au electrodes exhibited typical degradation behavior, characterized by a rapid decay in I_d_ and a significant negative shift in the onset voltage V_on_ over time. Notably, Au‐contact devices showed pronounced subthreshold slope broadening and progressive I_d_ decay; this was particularly evident in the low‐crystallinity P1, which suffered more severe degradation compared to its more ordered counterparts, P2 and P3. In stark contrast, devices incorporating Al/Ti electrodes demonstrated superior environmental stability, maintaining a substantial portion of their initial performance under identical conditions. To quantitatively evaluate these stability trends, we monitored the evolution of µ_sat_ and normalized mobility (µ_sat_ (t)/µ_sat_ (0)) over the seven‐day period (Figure 6d,e). After seven days, Au‐based devices lost approximately 80–90% of their initial mobility. Conversely, Al/Ti‐based devices effectively suppressed this degradation, with the mobility decrease limited to only 25–35%. Intriguingly, P1, which exhibited the most drastic performance drop with Au contacts falling from 0.041 to 0.003 cm^2^V^−^^1^s^−^^1^ (a 93% reduction), retained its performance most effectively with Al/Ti electrodes, decreasing only from 0.33 to 0.24 cm^2^V^−^^1^s^−^^1^ (a 25% reduction). These results confirm that the metal/semiconductor contact is a decisive factor in device stability and that proper contact engineering is essential for an accurate assessment of a material's intrinsic reliability. The stability of V_on_ also followed a similar trend. As shown in Figure 6f, Au‐contact devices exhibited substantial negative V_on_ shifts (ΔV_on_) of −32.8 V for P1, −17.8 V for P2, and −27.65 V for P3 after seven days of exposure. In contrast, Al/Ti‐contact devices showed significantly reduced V_on_ instability, with shifts of only −23.3 V (P1), −6.3 V (P2), and −14.84 V (P3) (See Section S7).

The operational reliability of the P1‐P3 series was further evaluated through continuous gate bias‐stress testing to determine the impact of electrode engineering on long‐term device stability. Figure 6g,h show the bias‐stress stability of P3 OFETs with Au and Al/Ti under continuous cycling (V_g_ = −10 to 100 V, V_d_ = 80 V), measured at day 0 (fresh) and after 4 days. For fresh OFETs, both Au and Al/Ti contacts exhibited highly stable operational characteristics, with negligible fluctuations in both on‐current and off‐current levels. However, a significant divergence in reliability emerged after 4 days of ambient exposure. In the case of Au‐contact devices, although the initial on‐current remained comparable to the fresh state, the current exhibited progressive decay and marked instability during repetitive on‐off cycling. This degradation suggests that the mismatched interface is more susceptible to charge trapping and electrical stress over time. In stark contrast, Al/Ti‐contact devices maintained remarkably stable on‐off current characteristics without any observable decay in the on‐current levels. Even after 7 days, despite a slight decrease in the absolute initial on‐current due to environmental aging, the operational stability during active cycling remained robust (See Supporting Information). The enhanced stability of Al/Ti‐contact devices likely originates from the reduced injection barrier and lower contact resistance, which suppress carrier accumulation at the source/drain interface [69, 70]. In Au‐contact devices, inefficient injection can lead to localized electric‐field concentration and prolonged carrier residence near the contact edge, promoting trap formation under ambient oxygen/moisture exposure and during bias stress [38, 45, 46, 47]. In contrast, the more ohmic Al/Ti interface enables more uniform charge injection and lowers interfacial stress [38, 72, 73], thereby mitigating trap generation and improving both environmental and operational stability. These results provide strong evidence that the metal/semiconductor contact characteristics play a decisive role in the electrical and environmental stability of the device, revealing impacts that extend far beyond their previously reported influence on charge injection efficiency [67, 68]. Specifically, the introduction of a suitable electrode such as Al/Ti for n‐type semiconductors facilitates a sustained channel‐limited transport regime. By minimizing the energetic barrier and stabilizing the interface, this approach effectively increases resistance to external trap formation, ensuring high‐fidelity operation even under prolonged stress in ambient conditions.

## 3 Conclusions

In conclusion, this study provides a comprehensive understanding of the interplay between semiconductor crystallinity and contact energetics in n‐type OFETs. By systematically evaluating a series of n‐type polymers (P1‐P3) with identical backbone chemistry but varying crystalline order, we demonstrated that the performance bottleneck in these devices is primarily governed by the metal/semiconductor interface rather than intrinsic bulk transport. Specifically, while all polymers exhibited remarkably similar intrinsic transport barriers of approximately 20 meV, their contact‐related energetics differed significantly, with lower crystallinity resulting in a more resistive injection environment. The implementation of low‐work‐function Al/Ti electrodes served as a decisive tool to decouple these effects, facilitating a transition from a contact‐limited to a channel‐limited transport regime. A pivotal finding of this work is the inverse correlation between semiconductor crystallinity and the magnitude of performance enhancement upon contact optimization; the least crystalline polymer (P1) showed a dramatic sevenfold increase in mobility, whereas the highly crystalline P3 exhibited more modest gains as it was already operating near its intrinsic transport limit. This suggests that relying on conventional Au electrodes can lead to a severe underestimation of the true potential of n‐type materials, particularly those with disordered morphologies. Furthermore, our results underscore the critical role of electrode engineering in enhancing environmental and operational stability. The Al/Ti contacts not only optimized charge injection but also provided an energetically and chemically robust interface that significantly suppressed trap formation and V_on_ instability under elevated relative humidity (45% RH) and continuous bias‐stress conditions. Ultimately, this research establishes a vital guideline for the organic electronics community: the accurate evaluation and optimization of n‐type semiconductors require a synergistic approach that accounts for the specific crystalline registry of the material. These insights provide a robust framework for the fair evaluation and rational optimization of high‐performance, air‐stable n‐type organic semiconductors.

## 4 Experimental

### 4.1 Materials

A series of n‐type thiophene‐flanked diketopyrrolopyrrole (DPP) copolymers were used as active channel layers: P2F2CNTVT–DPP 1 (P1), P2F2CNTVT–DPP 2 (P2), and P2F2CNTVT–DPP 3 (P3), differing systematically in their branched alkyl chains (P1: 2‐decyltetradecyl; P2: 2‐hexyltetradecyl; P3: 2‐octyldodecyl). Polymers were synthesized following a previously reported protocol [30]. All solvents, including 1,2‐dichlorobenzene and n‐butyl acetate, were procured from Sigma‐Aldrich and used as received. Poly(methyl methacrylate) (PMMA, 120 kDa) was also purchased from Sigma‐Aldrich and served as the gate dielectric.

### 4.2 Device Fabrication

Top‐gate, bottom‐contact (TG/BC) OTFTs were fabricated on Eagle glass substrates (Figure 2a). Substrates were ultrasonically cleaned by sequential rinsing and sonication in acetone (20 min), isopropanol (20 min), and deionized (DI) water (20 min) baths, followed by baking at 100°C in air for 20 min to remove residual organics. Forty‐nine transistors (7 × 7 array) were fabricated per substrate, with channel lengths of 50–350 µm and a channel width of 1200 µm. Patterned source–drain electrodes (Al/Ti: 20/5 nm or Au/Ni: 30/3 nm for control devices) were thermally evaporated through shadow masks under high vacuum (5 × 10^−^^4^ Pa). Polymer channel layers were spin‐coated from 1,2‐dichlorobenzene solutions (4 mg·mL^−^^1^) at 2000 rpm for 60 s inside a nitrogen‐filled glovebox, followed by annealing at 200°C for 1 h to enhance molecular packing and film uniformity. PMMA dielectric layers (80 mg·mL^−^^1^ in n‐butyl acetate) were spin‐coated in two steps (500 rpm for 3 s, then 2000 rpm for 60 s) and annealed at 80°C for 2 h. Finally, 80 nm‐thick Al top‐gate electrodes were thermally evaporated through shadow masks on top of the PMMA layer to complete the TG/BC architecture.

### 4.3 Characterization

Polymer layer thicknesses (P1–P3, ≈45–50 nm) and PMMA dielectric (≈500 nm) were measured using a surface profilometer (Bruker Dektak). Electrical current–voltage (I–V) measurements were performed in air (≈25°C, ≈45 % RH) using a probe station coupled to a Keysight B1500A semiconductor parameter analyzer. Low‐temperature I–V measurements were carried out from 95 K upward using a Keysight B1500A Semiconductor Parameter Analyzer (Center for Research Facilities (CRIEF) at Kongju National University). Capacitance–voltage (C–V) characteristics were recorded using a Keysight E4980A LCR meter. Molecular packing was characterized by grazing‐incidence wide‐angle X‐ray scattering (GIWAXS). GIWAXS measurements were performed at the Complex Materials Scattering (CMS) beamline of the National Synchrotron Light Source II (NSLS‐II), Brookhaven National Lab. The X‐ray beam with an energy of 10 keV shone upon the samples with an incident angle of 0.15 deg with respect to the substrate between the critical angles of the organic films and the Si substrate. A custom‐made Pilatus‐2 M detector was placed at a distance of 255 mm from the sample center to capture GIWAXS images with an exposure time of 10 s. Surface morphology was examined using atomic force microscopy (AFM, Bruker Dimension Icon), from which RMS roughness values were extracted to assess film uniformity. UV–vis (Digital TU1810 single‐beam UV–vis spectrophotometer, 190–1100 nm) spectroscopy was conducted on P1–P3 films to probe vibrational and optical properties. Ultraviolet photoelectron spectroscopy (UPS) measurements were performed using a Kratos Analytical AXIS Supra system equipped with a He I discharge source (E_ph_ = 21.22 eV) to determine the work function and energy‐level alignment of the deposited metals (Figure S4 and Section S3).

### 4.4 Extraction of Parameters

Key electrical parameters including threshold voltage (V_th_), subthreshold swing (SS), interface trap density (D_it_), turn‐on voltage (V_on_), saturation mobility (µsat), linear mobility (µ_lin_), contact resistance‐corrected linear mobility (µ_lin, corrected_), and activation energies (E_a_) from both mobility and contact resistance (R_c_) were extracted following standard procedures. For clarity, mobility‐ and interface‐related parameters were analyzed separately. Contact resistance and channel resistance were determined using the transfer line method (TLM) as a function of gate voltage (V_g_) and channel length (L), while the Transition Voltage Method (TVM) and Y‐Function Method were applied only for low‐temperature analysis. Detailed extraction procedures are provided in Sections S1 and S2.

## Author Contributions

**Chan Zhang**: methodology, investigation, conceptualization. **Jianeng Ma**: conceptualization, methodology, investigation. **Walid Boukhili**: writing – original draft, conceptualization, methodology, data curation. **Li Zhu**: investigation, formal analysis. **Changwoo Yu**: data curation, investigation. **Seung‐Hoon Lee**: conceptualization, methodology, validation, software, data curation. **Chee Leong Tan**: validation, investigation. **Dongyoon Khim**: writing – original draft, writing – review and editing, supervision, funding acquisition, project administration, conceptualization, methodology, visualization. **Zhuping Fei**: conceptualization, methodology, investigation, writing – review and editing, supervision. **Zhihao Yu**: investigation, validation, resources. **Huabin Sun**: investigation, validation. **Yong Xu**: validation. **Xiang Wan**: investigation, formal analysis, data curation. **Liyang Yu**: methodology, data curation. **Ruipeng Li**: methodology, data curation. **Guangan Yang**: investigation, formal analysis, data curation.

## Funding

This work was financially supported by NUPTSF (No. NY221113) and the Korea Basic Science Institute (National Research Facilities and Equipment Center) grant funded by the Korea Government (MSIT) (No. RS‐2024‐00403209 and RS‐2026‐25489371). L.Y. thanks the Fundamental Research Funds for the Central Universities for financial support. The authors also thank National Synchrotron Light Source II (NSLS‐II), (Contract No. DE‐ SC0012704) and Brookhaven National Laboratory for providing GIWAXS experiment time.

## Conflicts of Interest

The authors declare no conflicts of interest.

## Supporting information




**Supporting File**: advs77050‐sup‐0001‐SuppMat.docx.

## Data Availability

Research data are not shared.
