# Supplementary material for: Deciphering the Crystallinity‐Dependent Sensitivity of Charge Injection in n‐Channel Organic Transistors: Reliable Characterization and Optimized Performance
Source: Adv Sci (Weinh). 2026 Aug 21:e77050. Online ahead of print. doi: 10.1002/advs.77050 (PMC13496279; doi:10.1002/advs.77050)
Supplement: Supplementary file 1 [file ADVS-9999-e77050-s001.docx]

Supporting Information

**Deciphering the Crystallinity-Dependent Sensitivity of Charge Injection in n-Channel Organic Transistors**: **Reliable Characterization and Optimized Performance**

*Walid Boukhili, Chan Zhang, Jianeng Ma, Changwoo Yu, Chee Leong Tan, Huabin Sun, Zhihao Yu, Guangan Yang, Xiang Wan, Li Zhu, Yong Xu, Liyang Yu, Ruipeng Li, Seung-Hoon Lee*, Zhuping Fei* and Dongyoon Khim**

**Section 1**. Extraction of Electrical Parameters of P1-P3 OFETs

**Section 2.** Low-Temperature Transport Analysis and Contact Resistance Effects

**Section 3.** Al/Ti Electrode Engineering Enhances Electrical Properties in P1-P3 OFETs

**Section 4.** Effective Schottky Barrier Height Analysis

**Section 5.** Low-Temperature Electrical and Trap-State Analysis

**Section 6.** Ba(OH)_2_-Modified Au Electrodes: Interfacial Properties and OFET Performance

**Section 7.** Stability Enhancement in Al/Ti-Contact n-Type OFETs

**Section 1**

**Extraction of Electrical Parameters of P1- P3 OFETs**

Electrical parameters of P1-P3 OFETs were extracted from the transfer characteristics measured in the saturation regime (V_d_ = 80 V). The extracted parameters include saturation mobility (µ_sat_), linear mobility (µ_lin_), turn-on voltage (V_on_), on-current (I_on_), subthreshold swing (SS), and interface trap density (D_it_). Average values were obtained from 10 devices per polymer/contact configuration to ensure statistical reproducibility.

**Mobility Extraction:**

Linear and saturation mobilities were calculated using standard MOSFET equations.

Linear regime (V_d_ ≪ (V_g_ − V_th_)): ^[1-2]^

$$I_{d, lin}=\frac{W}{L}\mu_{lin}C_{i}\left( V_{g}-V_{th} \right)V_{d} (S1)$$

$$\mu_{lin}= \frac{L}{{WC}_{i}V_{d}}\left( \frac{\partial I_{d}}{\partial V_{g}} \right) (S2)$$

Saturation regime (V_d_ ≥ (V_g_ − V_th_)): ^[3]^

$$I_{d, sat}=\frac{W}{2L}\mu_{sat}C_{i}{(V_{g}-V_{th})}^{2} (S3)$$

$$\mu_{sat}=\frac{2L}{WC_{i}}\left( \frac{\partial\sqrt{I_{d}}}{\partial V_{g}} \right)^{2} (S4)$$

where W and L are the channel width and length, C_i_ is the gate dielectric capacitance per unit area, V_g_ is the gate voltage, and V_th_ is the threshold voltage.

**On-Current (I_on_):**

Defined as the maximum drain current extracted from the transfer characteristics at the applied gate and drain voltages.

**Turn-on Voltage (V_on_)**

Defined as the gate voltage at which the drain current begins to increase significantly from the off-state.

**Subthreshold Swing (SS)**: ^[3-4]^

$$SS=\frac{dV_{g}}{dlog\left( I_{d} \right)} (S5)$$

SS was extracted from the subthreshold region using small 3–5 point averaging windows to minimize noise. Each SS value was associated with the mean drain current of the corresponding window, and representative points were selected to construct the SS–I_d_ profile.

**Interface Trap Density (D_it_)**: ^[3-4]^

$$D_{it} =\left[ \frac{SS}{\frac{ln\left( 10 \right)k_{B}T}{q}}-1 \right]\frac{C_{i}}{q} (S6)$$

D_it_ was calculated from the measured SS values using Equation (S6), where k_B_ is the Boltzmann constant, T is the absolute temperature, and q is the elementary charge. D_it_ represents the interface trap density per unit area and energy.

**Figures S1a** and **S1b** show the Transfer Line Method (TLM) analysis in the linear regime (V_d_ = 5 V) for P1–P3 n-type OFETs with Au contacts. The plots display the width-normalized total resistance (R_tot_W) for multiple channel lengths (L = 50–350 µm, Vg = 40–80 V) and the corresponding channel (R_ch_W) and contact (R_c_W) resistances as functions of gate voltage.

**Figure S1.** Transfer Line Method (TLM) analysis in the linear regime (V_d_ = 5 V) for P1–P3 n-type OFETs with Au contacts. Panel (a) shows the width-normalized total resistance (R_tot_W) as a function of channel length (L) at gate voltages V_g_ = 30–80 V. Panel (b) displays the width-normalized channel resistance (R_ch_W) for selected channel lengths (L = 50–350 µm) and the width-normalized contact resistance (R_c_W) as a function of gate voltage.

**Section 2**

**Low-Temperature Transport Analysis and Contact Resistance Effects**

Low-temperature measurements were performed on polymer (P2 and P3) OFETs to probe intrinsic charge transport and the influence of contact resistance (R_c_) in OFETs. P1 was excluded from this analysis due to its significantly lower crystallinity.

**S2.1. Representative Device Characteristics**

Linear (V_d_ =5 V) and saturation (V_d_ =80 V) transfer characteristics of P2- and P3-OFETs measured at 95–289 K (**Figures S2a–S2b**) show that P2 exhibits broader subthreshold slopes and reduced on-currents at low temperatures, indicative of contact-limited charge transport caused by interfacial traps. In contrast, P3 maintains well-defined linear and saturation regimes, reflecting efficient injection and a higher molecular ordering.

**Figure S2.** Representative linear (V_d_= 5 V) and saturation (V_d_= 80 V) transfer characteristics measured at various low temperatures of (a) P2-OFETs and (b) P3-OFETs.

**S2.2. Contact Resistance Determination**:

R_c_ was quantitatively extracted using complementary methods to decouple contact and channel contributions. Both the Transition Voltage Method (TVM) and Y-function method were employed, showing strong agreement for all devices. Both methods agreed within ±5–14% for P2 and ±3–11% for P3, confirming robustness.

**TVM** [4]**:**

$R_{c}=2\frac{\sqrt{V_{g}-V_{th}} (\sqrt{2V_{tr}}-\sqrt{V_{g}-V_{th}} )}{I_{sat}} (S7)$

where V_th_ is the threshold voltage, V_tr_ is the transition voltage from linear to saturation regime, and I_sat_ is the saturation drain current.

**Y-function method:** ^[5]^

$Y\left( V_{g} \right)=\frac{I_{d}}{\sqrt{g_{m}}} (S8)$

with transconductance $g_{m}=\frac{\partial I_{d}}{\partial V_{g}}$ and gate-dependent mobility

$$\mu\left( V_{g} \right)=\frac{\mu_{0}}{1+\theta(V_{g}-V_{th})}$$

Linear-regime current including R_c_:

$I_{d}=\frac{K_{0}(V_{g}-V_{th})(V_{d}-I_{d}R_{c})}{1+\theta(V_{g}-V_{th})} (S9)$

where $K_{0}=\frac{W}{L}\mu_{0}C_{i}$. Then,

$R_{c}=\frac{V_{d}}{I_{d}}-\frac{L}{W\mu_{0}C_{i}\left( V_{g}-V_{th} \right)}-\frac{\theta L}{W\mu_{0}C_{i}} (S10)$

Here, μ_0_ is the intrinsic field-effect mobility and θ is the mobility attenuation factor associated with gate-voltage dependence. Parameters μ_0_, V_th_, and θ were extracted from linear fits of Y(V_g_) for all devices. Both methods consistently reveal strong thermal activation of R_c_ in P2 and weaker activation in P3.

Representative R_c_ at V_d_ = 5 V and V_g_ = 80  V:

**Table S1a.** The R_c_ of the P2 OFETs (Au contacts)

| T  (K) | R_c_ (TVM)  (kΩ·cm) | R_c_ (Y-function)  (kΩ·cm) | Δ  (%) |
| --- | --- | --- | --- |
| 291.15 | 3.46 × 10⁵ | 3.28 × 10⁵ | 5.2 |
| 273.15 | 4.61 × 10⁵ | 4.20 × 10⁵ | 8.9 |
| 253.15 | 6.46 × 10⁵ | 5.74 × 10⁵ | 11.2 |
| 229.15 | 9.03 × 10⁵ | 8.11 × 10⁵ | 10.2 |
| 213.15 | 1.28 × 10⁶ | 1.12 × 10⁶ | 12.5 |
| 193.15 | 1.89 × 10⁶ | 1.63 × 10⁶ | 13.8 |
| 173.15 | 2.99 × 10⁶ | 2.57 × 10⁶ | 14.0 |
| 155.15 | 4.43 × 10⁶ | 3.92 × 10⁶ | 11.5 |
| 135.15 | 5.76 × 10⁶ | 5.04 × 10⁶ | 12.5 |
| 112.15 | 8.49 × 10⁶ | 7.25 × 10⁶ | 14.6 |
| 95.15 | 1.18 × 10⁷ | 1.01 × 10⁷ | 14.2 |

**Table S1b.** The R_c_ of the P3 OFETs (Au Contacts)

| T  (K) | R_c_ (TVM)  (kΩ·cm) | Rc (Y-function) (kΩ·cm) | Δ  (%) | |
| --- | --- | --- | --- | --- |
| 289.15 | 1.68 × 10⁵ | 1.63 × 10⁵ | | 3.0 |
| 274.15 | 2.00 × 10⁵ | 1.92 × 10⁵ | | 4.0 |
| 254.15 | 2.28 × 10⁵ | 2.15 × 10⁵ | | 5.7 |
| 233.15 | 2.67 × 10⁵ | 2.44 × 10⁵ | | 8.6 |
| 213.15 | 3.38 × 10⁵ | 3.07 × 10⁵ | | 9.0 |
| 193.15 | 4.27 × 10⁵ | 3.83 × 10⁵ | | 10.4 |
| 174.15 | 4.76 × 10⁵ | 4.33 × 10⁵ | | 9.0 |
| 154.15 | 5.59 × 10⁵ | 5.09 × 10⁵ | | 8.9 |
| 133.15 | 6.56 × 10⁵ | 5.88 × 10⁵ | | 10.4 |
| 113.15 | 8.13 × 10⁵ | 7.26 × 10⁵ | | 10.7 |

Each value represents the mean of 10 devices; deviations correspond to ±1 σ, ensuring statistical reliability.

**S2.3. Linear Mobility Correction for R_c_**

To isolate intrinsic transport, the effective channel voltage is defined as: ^[6]^

$${V_{d,eff}=V}_{d}-{R_{c}I}_{d} (S11)$$

Corrected linear current:

$$I_{d}=\left( \frac{W}{L} \right){C_{i} \mu}_{lin, corr}\left( V_{g}-V_{th} \right)\left( V_{d}-{R_{c}I}_{d} \right) (S12)$$

Hence,

$$\mu_{lin, corr}=\left[ \frac{L}{{WC}_{i}\left( V_{d}-{R_{c}I}_{d} \right)} \right]\left[ \frac{\partial I_{d}}{\partial V_{g}} \right]_{V_{d=cte}} (S13)$$

Correction is essential in the linear regime where R_c_ dominates.

**Table S2a.** P2 Linear Mobility Correction

| 1000/T (K^-1^) | μ_lin, uncorrected_  (cm²V⁻¹s⁻¹) | μ_lin, corrected_  (cm²V⁻¹s⁻¹) | Mobility ideal factor  (μ_corr_ / μ_uncorr_) |
| --- | --- | --- | --- |
| 3.43466 | 0.14024 | 0.205 | 1.46 |
| 3.66099 | 0.11962 | 0.18 | 1.50 |
| 3.95023 | 0.09152 | 0.165 | 1.80 |
| 4.36395 | 0.07058 | 0.134 | 1.90 |
| 4.69153 | 0.04805 | 0.128 | 2.66 |
| 5.17732 | 0.04115 | 0.111 | 2.70 |
| 5.77534 | 0.03534 | 0.098 | 2.77 |
| 7.39919 | 0.02886 | 0.069 | 2.39 |

**Table S2b.** P3 Linear Mobility Correction

| 1000/T (K^-1^) | μ_lin, uncorrected_  (cm²V⁻¹s⁻¹) | μ_lin, corrected_  (cm²V⁻¹s⁻¹) | Mobility ideal factor  (μ_corr_ / μ_uncorr_) |
| --- | --- | --- | --- |
| 3.45841 | 0.57159 | 0.678 | 1.19 |
| 3.64764 | 0.51908 | 0.605 | 1.17 |
| 3.93468 | 0.46760 | 0.542 | 1.16 |
| 4.28908 | 0.38132 | 0.474 | 1.24 |
| 4.69153 | 0.30757 | 0.445 | 1.45 |
| 5.17732 | 0.24177 | 0.400 | 1.65 |
| 5.74218 | 0.22650 | 0.355 | 1.57 |
| 7.51033 | 0.18322 | 0.278 | 1.52 |

P2 shows strong μ_lin_ suppression due to large R_c_; P3 maintains higher μ_lin_, confirming channel-limited transport. Correction factors are 1.46–2.77× for P2 and 1.16–1.65× for P3.

**S2.4. Activation Energies (E_a_)**

Mobility follows Arrhenius dependence: ^[7]^

$$\mu\left( T \right)=\mu_{0}\exp\left( -\frac{E_{a}}{k_{B}T} \right) (S14)$$

E_a_ (μ_lin, uncorr_): uncorrected linear mobility

E_a_ (μ_lin, Rc-corr_): R_c_-corrected linear mobility

E_a_ (μ_sat_): saturation mobility

Contact resistance is also thermally activated: ^[7]^

$R_{c}\left( T \right)=R_{0}\exp\left( \frac{E_{a}}{k_{B}T} \right) (S15)$

Correcting for R_c_ reduces E_a_ (μ_lin_) from ~34.5 to 22.4 meV in P2, approaching E_a_ (μ_sat_) ~20.2 meV. P3 reduction is modest (25.5 to 18.2 meV). R_c_ activation energies: 63.1 meV (P2) and 29 meV (P3).

This activation reflects thermally assisted charge injection across interfacial barriers combined with hopping transport through localized states in the contact access region. All activation energies were extracted from Arrhenius plots (ln(μ) vs 1/T and ln (R_c_) vs 1/T) in the 95–290 K range.

**Table S3:** Activation energy and R_c_ summary

| Polymer | E_a_ (μ_lin, uncorr_)  (meV) | E_a_ (μ_lin, Rc-corr_) (meV) | E_a_(μ_sat_) [meV] | R_c_  (kΩ·cm) | E_a_ (R_c_)  (meV) |
| --- | --- | --- | --- | --- | --- |
| P2 | 34.5 ± 2.1 | 22.4 ± 1.3 | 20.2 ± 1.2 | 10²–10⁴ | 63.1 ± 3.5 |
| P3 | 25.5 ± 1.8 | 18.2 ± 1.1 | 17.2 ± 0.9 | 10–10² | 29.0 ± 2.1 |

Correlation of E_a_ (μ_lin_) and E_a_ (R_c_) confirms that injection, not intrinsic channel transport, dominates at low temperature in disordered polymers.

**S2.5. Discussion**

Low-temperature transport of P2 and P3 OFETs illustrates the influence of polymer crystallinity and contact interface. R_c_ extraction via TVM and Y-function agreed within ±5-14 % (P2) and ±3-11 % (P3), confirming robustness. Linear mobility (μ_lin_) was corrected using the effective channel voltage, yielding μ_lin, corr_ that approaches μ_sat_ and reduces the E_a_ from ~34.5 to 22.4 meV in P2; P3 shows modest correction (factor 1.16-1.65×, E_a_ ~18.2 meV). Arrhenius analysis of both μ_lin, corr_ and μ_sat_ reveals higher thermal activation in P2 due to hopping across disordered states and interface barriers, while P3 exhibits lower disorder and shallower traps (E_a_(μ_sat_) ~17.2 meV). These results confirm that accurate R_c_ extraction and linear mobility correction are essential to isolate intrinsic transport properties, quantify thermal activation, and guide rational design of polymer backbones and contact interfaces for optimized OFET performance.

**Section 3**

**Al/Ti Electrode Engineering Enhances Electrical Properties in P1-P3 OFETs**

**S3.1. Transfer Line Method (TLM) analysis results**

Al/Ti bilayer electrodes significantly enhance n-type OFET performance across low- and high-crystallinity polymers by reducing injection barriers, lowering contact resistance, promoting efficient charge injection, and improving environmental and operational stability.

**Figures S3a** and **S3b** show the Transfer Line Method (TLM) analysis in the linear regime (V_d_= 5 V) for P1–P3 n-type OFETs with Al/Ti contacts, to quantify contact and channel resistances. The plots show the width-normalized total resistance (R_tot_W) for multiple channel lengths (L = 50–350 µm, Vg = 40–80 V) (**Figure S3a**), along with the width-normalized channel resistance (R_ch_W) and contact resistance (R_c_W) as functions of gate voltage (**Figure S3b**).

**Figure S3.** Transfer Line Method (TLM) analysis in the linear regime (Vd = 5 V) for P1–P3 n-type OFETs with Al/Ti contacts. Panel (a) shows the width-normalized total resistance (R_tot_W) as a function of channel length (L) at gate voltages Vg = 30–80 V. Panel (b) shows the width-normalized channel resistance (R_ch_W) for selected channel lengths (L = 50–350 µm) and the width-normalized contact resistance (R_c_W) as a function of gate voltage.

**S3.2. Work Function (WF) Measurements of Au and Al/Ti Electrodes**

The work functions of Au and Al/Ti electrodes were measured using ultraviolet photoelectron spectroscopy (UPS). **Figures S4a** and **S4b** show the UPS spectra for Au (panel a) and Al/Ti (panel b), respectively.

**Figure S4.** UPS spectra of (a) Au and (b) Al/Ti electrodes, with the formula used to determine the work function (WF) indicated.

The work function was calculated using the standard relation: ^[8-9]^

WF = hν – (E_cutoff_ –E_F_)

where hν = 21.22 eV corresponds to the He I excitation energy, and E_cutoff_ and E_F_ denote the binding energies of the secondary electron cutoff and the Fermi level, respectively.

Using E_F_ = 0 eV as reference, WF(Au) ≈ 4.5 eV and WF(Al/Ti) ≈ 4.0 eV.

These measured work functions were then used to estimate the corresponding Schottky barrier heights (Φ_B_) at the polymer–electrode interface for P1–P3 OFETs. The lower WF of Al/Ti electrodes effectively reduces the injection barrier, facilitating electron injection and improving charge transport, particularly in low-crystallinity polymers.

**Section 4**

**Effective Schottky Barrier Height Analysis**

Although charge transport in organic semiconductors occurs via hopping, the thermionic-emission formalism provides an effective framework for comparing injection barriers under different contact conditions. In OFETs, R_c_ generally comprises contributions from both the intrinsic metal/semiconductor interface (R_int_) and the access region in the semiconductor adjacent to the contacts (R_acc_). ^[10-12]^ In this analysis, we do not separate these components explicitly. R_int_ is generally governed by the Schottky barrier, determined by the band alignment of the electrode and semiconductor as well as any doping, whereas R_acc_ originates from the semiconductor bulk, including factors such as film thickness, crystallinity, and energetic disorder. ^[12]^ Because R_int_ is governed by the Schottky barrier, the following analysis focuses on the effective Schottky barrier (Φ_B, eff_). (**Figure S5**). To elucidate the possible origin of the differences in R_c_ between P2 and P3 devices, we compare activation energies extracted from mobility and contact-resistance analyses with the effective Schottky barrier (Φ_B, eff_) (**Figure 4e, Table S5**). E_a_ (R_c_) reflects the total R_c_, while Φ_B,_ _eff_, extracted from ln (I_d_/T²) versus q/(k_B_T) ^[11]^ (**Figure 4b, Figure S5 (g, h)**), represents the field-lowered injection barrier under bias and thus implicitly accounts for the influence of R_acc_. ^[11]^ Because R_c_ = R_int_ + R_acc_, one expects Φ_B, eff_ ≤ E_a_ (R_c_), and the difference between these quantities quantifies the additional activation barrier associated with transport through the semiconductor bulk. For the highly crystalline polymer P3, this difference is small: E_a_ (R_c_)≈ 29 meV and Φ_B,_ _eff_ ≈ 27 meV, indicating that R_acc_ is minimal due to efficient π–π stacking, low energetic disorder, and high local mobility near the contacts. Consequently, charge injection is dominated by the interfacial barrier. In contrast, P2 exhibits a pronounced disparity between E_a_ (R_c_) ≈ 63 meV and Φ_B_, _eff_ ≈ 57 meV, demonstrating that the access resistance (R_acc_) is significant in the lower-crystallinity P2 polymer. The results indicate that the Schottky barrier appears more pronounced in the lower-crystallinity P2 devices. Although P2 and P3 have similar LUMO levels, the contact resistance of P2 is higher due to its lower crystallinity and higher trap density. These findings indicate that contact-limited behavior is strongly influenced by semiconductor crystallinity, which strongly modulates R_acc_ and the overall R_c_, exerting a greater influence than the energy level alignments on device performance.

The effective Schottky barrier height (Φ_B, eff_) was quantitatively determined based on the thermionic-emission formalism: ^[13-15]^

$$I_{d}=AA^{*}T^{2}exp\left( -\frac{q\Phi_{B, eff}}{k_{B}T} \right) \left( S16 \right)$$

where A is the contact area and A^⁎^ is the Richardson constant. Φ_B, eff_ was obtained by performing linear fits to Arrhenius plots of ln(I_d_/T²) versus q/(k_B_T), for which the slope directly yields − Φ_B, eff_. Φ_B, eff_ represents an apparent barrier including image-force and field-assisted lowering under applied bias.

The Arrhenius data (**Figure 4b** (V_d_ = 5 V) and **Figure S5 (g, h)** (V_d_ = 5–80 V)), taken from the temperature-dependent output curves, show clear thermally activated behavior, enabling reliable extraction of Φ_B, eff_. The Φ_B, eff_ calculated here is a barrier deformed by the electric field rather than its intrinsic value and includes the energy barrier that charge carriers need to overcome in the polymer bulk.

The resulting Φ_B, eff_ for P2 and P3, plotted as a function of drain voltage at V_g_ = 80 V in **Figure 4c**, reveal systematically lower Φ_B, eff_ for the more crystalline P3 polymer. In contrast, P2 exhibits higher apparent barrier values across the entire bias range, reflecting less efficient injection in the low-crystallinity semiconductor.

**Figure S5.** Temperature-dependent electrical characteristics and contact resistance. **(a, b)** Output characteristics of P2- and P3-based OFETs measured at various temperatures. **(c, d)** Corresponding output curves in the low-V_D_ regime at V_G_ = 80 V. These data were used to construct the Arrhenius plots for Schottky barrier extraction. Temperature dependence of the channel-width-normalized contact resistance extracted at various gate voltages for **(e)** P2- and **(f)** P3-based devices. Arrhenius fits of ln(Id/T²), where I_d_ is obtained from the output curves, plotted versus q/(k_B_T) for **(g)** P2 and **(h)** P3 devices.

**Table S4.** Summary of Activation Energies and Effective Schottky Barrier Height with Their Physical Significance for P2 and P3 OFET Devices.

| Parameter | Definition / Physical Meaning |
| --- | --- |
| E_a_ (µ_lin_) (meV) | Activation energy of linear-regime mobility; includes both intrinsic transport and contact effects |
| E_a_ (µ_lin, corrected_) | Linear mobility activation energy after correcting for total contact resistance; reflects intrinsic channel transport |
| E_a_ (µ_sat_) | Activation energy of saturation-regime mobility; less sensitive to contact resistance than μlin and more representative of channel transport. |
| E_a_ (R_c_) | Activation energy of total contact resistance Rc; includes both interfacial injection barrier and access-region contribution |
| Φ_B, eff_ (ln (I_d_/T²) method) (meV) | Effective Schottky barrier at the metal/semiconductor interface; isolates the injection barrier from bulk access effects |

**Section 5**

**Low-Temperature Electrical and Trap-State Analysis**

This section presents a detailed analysis of top-gate/bottom-contact OFETs (P2 and P3) under low-temperature conditions, focusing on threshold voltage (V_th_) and trapped charge (N_trap_), drain current (I_d_) and its temperature dependence (activation energy, E_a_), and the density-of-states (DOS).

The presence of charge trapping is clearly evident in the temperature-dependent behavior of threshold voltage (V_th_) for P2 (low-crystallinity) and P3 (high-crystallinity) OFETs. V_th_ shifts to more positive values as temperature decreases due to increased carrier trapping and reduced thermal emission from localized states. At higher temperatures, thermally activated de-trapping lowers the net trapped charge, resulting in a reduced threshold voltage.

To quantify this effect, the threshold voltage shift (ΔV_th_) is defined as:

ΔV_th_ (T)=V_th_ (T) − V_th_ (T_max_)

where T_max_ corresponds to the maximum measured temperature. ΔV_th_ is temperature-dependent due to trap occupancy changes.

The broad temperature coverage and low device-to-device variability in our measurements allow a clear quantitative correlation between ΔV_th_ and trap-limited mobility.

The maximum areal trap density estimated from the subthreshold swing, $N_{SS}^{\max}$, can be determined from using the established relation:

$$N_{SS}^{\max}=\frac{C_{i}}{q} \left[ \frac{SS}{\frac{ln\left( 10 \right)k_{B}T}{q}}-1 \right] (S17)$$

ΔV_th_ can then be converted to the corresponding areal trapped charge density ΔN_trap_ using Equation S18:

$$\Delta N_{trap}\left( T \right)=\frac{C_{i}}{q}\Delta V_{th}\left( T \right) \left( S18 \right)$$

Equation (S17) describes the charge trap density derived from SS, while Equation (S18) converts ΔV_th_ to an equivalent trapped charge density ΔN_trap_.

By applying these two relations, the trapped charge N_trap_ can be calculated at each temperature.

**Determination of the Density of States (DOS)**

The density of states (DOS) is determined using the relation: ^[16-18]^

$$DOS\left( E \right)=(\frac{C_{i}}{qt}) \left| \frac{{dE}_{a}}{{dV}_{g}} \right|^{-1} (S19)$$

where C_i_=3.5×10^−9^ F/cm^2^ is the capacitance of the PMMA dielectric per unit area, E_a_ is the activation energy of transport, t is the effective accumulation layer thickness, approximated here by the semiconductor film thickness as an upper-limit estimate. This provides an upper-limit estimate of the trap DOS since the accumulation layer is typically confined near the dielectric interface.

This relation assumes that the Fermi level shifts linearly with gate voltage and that the accumulated charge is uniformly distributed within an effective accumulation layer of thickness t. Here DOS is expressed as a volume trap density assuming uniform vertical carrier distribution within thickness t.

Intuitively, a rapid variation of E_a_ with V_g_ indicates that the Fermi level easily shifts toward the band edge, corresponding to a low density of localized trap states. Conversely, a high DOS leads to smaller changes in E_a_ with V_g_, reflecting the presence of abundant traps.

To determine the activation energy (E_a_) as a function of gate voltage, we first consider the dependence of the drain current I_d_ on the channel conductivity σ at a fixed drain voltage V_d_: ^[16-18]^

$$I_{d}=\frac{W}{L}t\sigma V_{d} (S20)$$

where tσ represents the effective sheet conductance of the accumulated channel.

The conductivity follows a thermally activated behavior according to: ^[16-18]^

$$\sigma=\sigma_{0} {exp}^{(\frac{{-E}_{a}}{k_{B}T})} (S21)$$

Plotting ln(I_d_) versus 1000/T (Arrhenius plot) at fixed V_g_ yields a linear slope from which the activation energy E_a_ can be extracted (**Figure S6 a, b**).The extracted E_a_ as a function of gate voltage (**Figure S6 c**) shows a systematic decrease with increasing V_g_, reflecting the progressive filling of shallow (band-tail) and deep traps in the polymer.

Then, applying Eq. S19, we can calculate the DOS for each polymer (**Figure S6 d**).

This analysis allows direct comparison of shallow and deep trap distributions in P2 and P3, correlating the differences in threshold voltage (V_th_), trapped charge (N_trap_), and drain current (I_d_) with the energetic distribution of localized states in the channel.

**Figure S6.** Low-temperature electrical and trap-state analysis of P2 and P3 OFETs. (a, b) Arrhenius plots of the drain–source current (I_d_) versus 1000/T for (a) P2 and (b) P3 devices in the saturation regime at fixed V_d_. Linear fits (solid lines) are used to extract the activation energies. (c) Gate voltage (V_g_) dependence of the activation energy (E_a_) for P2 and P3, obtained from the slopes of the Arrhenius plots. (d) Corresponding trap density-of-states (DOS, cm⁻³ eV⁻¹) spectra calculated from E_a_(V_g_) using Eq. S19, highlighting the significantly reduced shallow band-tail DOS in P3 near the transport level, while the deep trap densities at larger activation energies remain comparable for both polymers.

**Table S5.** Classification of Trap States and Their Impact on OFET Performance

| Trap type | Energetic position | Main device impact |
| --- | --- | --- |
| Shallow traps (band-tail states) | Close to the transport level (band tail) | Govern field-effect mobility, mobility activation energy, and contact resistance through multiple trapping-and-release processes |
| Deep traps | Energetically far from the transport level | Influence threshold voltage, bias stability, and hysteresis, with minimal direct impact on mobility |

The trap density-of-states (DOS) spectra obtained at low temperature show that P2 and P3 exhibit comparable densities of deep trap states, whereas P3 possesses a markedly lower shallow band-tail DOS. This indicates that the performance disparity between the two polymers is not caused by deep traps, but rather by the reduced density of shallow band-tail states located close to the transport level. The lower shallow-trap density in P3 reduces energetic disorder and suppresses multiple trapping-and-release events near the injecting contacts, thereby lowering the apparent (effective) contact resistance and enabling significantly higher field-effect mobility in top-gate/bottom-contact OFETs with Au electrodes.

**Section 6**

**Ba(OH)_2_-Modified Au Electrodes: Interfacial Properties and OFET Performance**

To further clarify the role of contact engineering across semiconductors with different microstructures, we introduced a solution-processed Ba(OH)_2_ interlayer at the Au/semiconductor interface. This experiment was designed to verify whether the contact-modulation effect observed for Al/Ti electrodes is a general interfacial phenomenon or remains dependent on the intrinsic structural order of the polymer. By modifying the Au work function without altering the semiconductor energy levels, this approach enables direct evaluation of how microstructure influences charge injection and contact resistance.

*Fabrication of OFETs with Ba(OH)_2_-Modified Au Electrodes:* Top-gate, bottom-contact (TG/BC) organic field-effect transistors incorporating Ba(OH)_2_-modified Au source/drain electrodes were fabricated on Eagle glass substrates following the same baseline procedure as the control devices. After substrate cleaning and patterning of Au source/drain electrodes (30/3 nm Au/Ni) by thermal evaporation through shadow masks (5 × 10⁻⁴ Pa), a barium hydroxide interfacial layer was introduced prior to semiconductor deposition. Ba(OH)_2_ (Sigma-Aldrich) was dissolved in methanol at a concentration of 2 mg mL⁻¹ and filtered before use. The solution was spin-coated onto the patterned Au electrodes at 4000 rpm for 30 s under ambient conditions, forming an ultrathin electron-injection interlayer. Following interlayer formation, polymer semiconductor films were spin-coated from 1,2-dichlorobenzene solutions (4 mg mL⁻¹) at 2000 rpm for 60 s inside a nitrogen glovebox and annealed at 200 °C for 1 h. A PMMA dielectric layer (80 mg mL⁻¹ in n-butyl acetate) was then deposited via a two-step spin process (500 rpm for 3 s, 2000 rpm for 60 s) and annealed at 80 °C for 2 h. Finally, 80 nm Al top-gate electrodes were thermally evaporated through a shadow mask to complete the TG/BC device architecture.

**Figure S7** demonstrates that introducing a Ba(OH)_2_ interlayer at the Au/semiconductor interface leads to systematic improvements in OFET performance for all three polymers, despite their comparable LUMO energy levels. AFM and UPS measurements confirm that Ba(OH)_2_ forms nanoscale aggregates on Au and reduces the electrode work function to approximately 4.1 eV, facilitating electron injection. Notably, the extent of mobility enhancement varies significantly among P1–P3, with the largest relative gains observed for the less crystalline polymers (**Table S6**). These results indicate that the effectiveness of contact modification is strongly modulated by polymer microstructure rather than energy level alignment alone, consistent with trends observed for Al/Ti electrodes.

**Figure S7.** Characterization of Ba(OH)_2_-modified Au electrodes and corresponding OFET performance.

(a) Schematic illustration of the TG/BC OFET architecture incorporating a Ba(OH)₂ interlayer between the Au source/drain electrodes and the semiconductor. (b) AFM topography of Ba(OH)₂ deposited on an Au substrate, showing the formation of aggregated nanoscale domains. (c) Ultraviolet photoelectron spectroscopy (UPS) spectrum of the Au/Ba(OH)_2_ surface, indicating a reduced work function to 4.1 eV. (d) Transfer characteristics of OFETs based on P1 (blue), P2 (red), and P3 (cyan) polymers with Ba(OH)_2_ interlayers. The extracted saturation mobilities (μ_sat_) are indicated in the respective plots.

**Table S6.** Comparison of saturation electron mobility before and after Ba(OH)_2_ interlayer insertion at Au contacts, highlighting microstructure-dependent performance enhancement in P1–P3 OFETs.

| Polymer | μ_sat_ (Au)  (cm²V⁻¹s⁻¹) | μ_sat_ (Au/Ba(OH)_2_)  (cm²V⁻¹s⁻¹) | Enhancement Factor |
| --- | --- | --- | --- |
| P1 | 0.065 | 0.43 | 6.6× |
| P2 | 0.20 | 1.0 | 5× |
| P3 | 0.85 | 1.1 | 1.3× |

The trends observed with Ba(OH)_2_-modified Au contacts closely mirror those obtained with Al/Ti electrodes, where larger performance gains are found in the less crystalline polymers. This consistency supports the conclusion that interfacial contact resistance, rather than energy level alignment alone, governs injection efficiency in these systems.

**Section 7**

**Stability Enhancement in Al/Ti-Contact n-Type OFETs**

The long-term operational stability of n-type OFETs based on P1–P3 polymers was systematically evaluated under elevated relative humidity (45% RH). Devices incorporating Al/Ti bilayer electrodes were compared to Au-contact counterparts. All parameters were averaged over 10 devices per polymer/contact configuration, ensuring statistical reliability. The extracted metrics include mobility (µ_sat_) retention, on-current (I_on_) retention, turn-on voltage (V_on_) shift, and bias-stress stability.

The summarized results in **Table S7** confirm the superior performance retention of Al/Ti-contact devices compared to Au-contact counterparts across all polymers, particularly in low-crystallinity systems.

**Table S7.** Quantitative comparison of µsat retention, Ion retention, Von shift, and bias-stress stability in P1–P3 OFETs with Au and Al/Ti electrodes under ~45 % RH. Each value is the average of 10 devices per polymer/contact configuration.

| Polymer | Contact | Mobility Retention  (After 7 days) | I_on_ Retention  (After 7 days) | V_on_ Shift  (After 7 days) | Bias-Stress Stability |
| --- | --- | --- | --- | --- | --- |
| P1 | Au | ~7 % | ~3 % | −32.8 ± 1.2 V | Poor |
| P1 | Al/Ti | ~73 % | ~26 % | −23.3 ± 1.1 V | Excellent |
| P2 | Au | ~20.5 % | ~5 % | −17.8 ± 0.9 V | Moderate |
| P2 | Al/Ti | ~62.5 % | ~24 % | −6.3 ± 0.4 V | Excellent |
| P3 | Au | ~15.9 % | ~4 % | −27.65 ± 1.3 V | Moderate |
| P3 | Al/Ti | ~63 % | ~19 % | −14.84 ± 0.7 V | Excellent |

Al/Ti bilayer electrodes enhance n-type OFET stability by lowering contact resistance, suppressing interfacial traps, and maintaining efficient charge injection. Even low-crystallinity P1 retains ~73 % of its initial mobility with excellent bias-stress stability, while P2 and P3 show minimal ΔV_on_ and stable operation.

Enhanced stability arises from favorable Al/Ti–LUMO alignment, Ti buffer-mediated interface passivation, and optimized polymer microstructural order. Collectively, these effects suppress carrier scattering and trap formation, ensuring robust, channel-limited transport, highlighting Al/Ti contacts as an effective strategy for improving performance and operational durability in n-type OFETs.

**References**

[1] G. Horowitz, R. Hajlaoui, D. Fichou, and A. El Kassmi, “Gate voltage dependent mobility of oligothiophene field-effect transistors,” Journal of Applied Physics 85 (1999): 3202–3206. <https://doi.org/10.1063/1.369661>.

[2] R. Bourguiga, F. Garnier, G. Horowitz, R. Hajlaoui, P. Delannoy, M. Hajlaoui, and H. Bouchriha, “Simulation of I–V characteristics of organic thin film transistor: Application to the dihexylquaterthiophene,” The European Physical Journal Applied Physics 14 (2001): 121. <https://doi.org/10.1051/epjap:2001146>.

[3] W. Boukhili, M. Mahdouani, M. Erouel, J. Puigdollers, and R. Bourguiga, “Reversibility of humidity effects in pentacene based organic thin-film transistor: Experimental data and electrical modeling,” Synthetic Metals 199 (2015): 303–309. <https://doi.org/10.1016/j.synthmet.2014.12.009>.

[4] S. Wageh, W. Boukhili, and A. Al-Ghamdi, “Negative differential resistance behavior in n-channel organic thin-film transistors based on C60 and PTCDI-C8: Electrical characterization and parameter extraction,” Physica Status Solidi A 219 (2022): 2100500. <https://doi.org/10.1002/pssa.202100500>.

[5] W. Boukhili, S.-H. Lee, Q. Chen, X. Wan, C. L. Tan, H. Sun, Z. Yu, Y. Xu, K.-J. Baeg, B.-G. Kang, and D. Khim, “Gate dielectric effects on ambipolar transport in polymer-wrapped single-walled carbon nanotube network transistors,” ACS Applied Electronic Materials 7 (2025): 6051–6061. <https://doi.org/10.1021/acsaelm.5c00747>.

[6] A. Al-Ghamdi, W. Boukhili, and S. Wageh, “Contact resistance-corrected electrical characteristics with channel length effects in π-conjugated small-molecule benzanthracene organic thin film transistors,” Synthetic Metals 273 (2021): 116670. <https://doi.org/10.1016/j.synthmet.2020.116670>.

[7] R. J. Chesterfield, J. C. McKeen, C. R. Newman, C. D. Frisbie, P. C. Ewbank, K. R. Mann, and L. L. Miller, “Variable temperature film and contact resistance measurements on operating n-channel organic thin film transistors,” Journal of Applied Physics 95 (2004): 6396–6405. <https://doi.org/10.1063/1.1710729>.

[8] W. Song and M. Yoshitake, “A work function study of ultra-thin alumina formation on NiAl(110) surface,” Applied Surface Science 251 (2005): 14–18. <https://doi.org/10.1016/j.apsusc.2005.03.116>.

[9] Y. Park, V. Choong, Y. Gao, B. R. Hsieh, and C. W. Tang, “Work function of indium tin oxide transparent conductor measured by photoelectron spectroscopy,” Applied Physics Letters 68 (1996): 2699–2701. <https://doi.org/10.1063/1.116313>.

[10] T. Minari, T. Miyadera, K. Tsukagoshi, Y. Aoyagi, and H. Ito, “Charge injection process in organic field-effect transistors,” Applied Physics Letters 91 (2007): 053508. <https://doi.org/10.1063/1.2759987>.

[11] Q. Wang, S. Jiang, B. Zhang, E.-Y. Shin, Y.-Y. Noh, Y. Xu, Y. Shi, and Y. Li, “Role of Schottky barrier and access resistance in organic field effect transistors,” The Journal of Physical Chemistry Letters 11 (2020): 1466–1472. <https://doi.org/10.1021/acs.jpclett.9b03339>.

[12] S. D. Wang, T. Minari, T. Miyadera, K. Tsukagoshi, and Y. Aoyagi, “Contact-metal dependent current injection in pentacene thin-film transistors,” Applied Physics Letters 91 (2007): 203508. <https://doi.org/10.1063/1.2813640>.

[13] E. H. Rhoderick, “The physics of Schottky barriers,” Review of Physics in Technology 1 (1970): 115–167. <https://doi.org/10.1088/0034-6683/1/2/302>.

[14] A. Shehu, S. D. Quiroga, P. D’Angelo, C. Albonetti, F. Borgatti, M. Murgia, A. Scorzoni, P. Stoliar, and F. Biscarini, “Layered distribution of charge carriers in organic thin film transistors,” Physical Review Letters 104 (2010): 246602. <https://doi.org/10.1103/PhysRevLett.104.246602>.

[15] Y. Xu, H. Sun, W. Li, Y.-F. Lin, F. Balestra, G. Ghibaudo, and Y.-Y. Noh, “Exploring the charge transport in conjugated polymers,” Advanced Materials 29 (2017): 1702729. <https://doi.org/10.1002/adma.201702729>.

[16] J. Puigdollers, A. Marsal, S. Cheylan, C. Voz, and R. Alcubilla, “Density of states in pentacene from the electrical characteristics of thin-film transistors,” Organic Electronics 11 (2010): 1333–1337. <https://doi.org/10.1016/j.orgel.2010.05.007> .

[17] T. Globus, H. C. Slade, M. Shur, et al., “Density of deep bandgap states in amorphous silicon from the temperature dependence of thin film transistor current,” MRS Online Proceedings Library 336 (1994): 823–828. <https://doi.org/10.1557/PROC-336-823>.

[18] D. V. Lang, X. Chi, T. Siegrist, A. M. Sergent, and A. P. Ramirez, “Amorphous-like density of gap states in single-crystal pentacene,” Physical Review Letters 93 (2004): 086802. <https://doi.org/10.1103/PhysRevLett.93.086802>.
